# Comparing the Efficacy of CT, MRI, PET-CT, and US in the Detection of Cervical Lymph Node Metastases in Head and Neck Squamous Cell Carcinoma with Clinically Negative Neck Lymph Node: A Systematic Review and Meta-Analysis

**DOI:** 10.3390/jcm13247622

**Published:** 2024-12-14

**Authors:** Ahmed Alsibani, Abdulwahed Alqahtani, Roaa Almohammadi, Tahera Islam, Mohammed Alessa, Saleh F. Aldhahri, Khalid Hussain Al-Qahtani

**Affiliations:** 1 Department of Otolaryngology-Head & Neck Surgery, King Saud University, Riyadh 11411, Saudi Arabia; abualwaleed1402@gmail.com (A.A.); maalessa@ksu.edu.sa (M.A.); kalqahtani@ksu.edu.sa (K.H.A.-Q.); 2 College of Medicine, Taibah University, Madinah 42311, Saudi Arabia; 3 College of Medicine and Research Center, King Saud University, Riyadh 11411, Saudi Arabia; tahera@ksu.edu.sa

**Keywords:** cervical lymph node metastases, HNSCC, CT, MRI, PET-CT, ultrasound

## Abstract

**Background:** Traditional imaging techniques have limited efficacy in detecting occult cervical lymph node (LN) metastases in head and neck squamous cell carcinoma (HNSCC). Positron emission tomography/computed tomography (PET-CT) has demonstrated potential for assessing HNSCC, but the literature on its efficacy for detecting cervical LN metastases is scarce and exhibits varied outcomes, hindering comparisons. **Aim:** To compare the efficacy of CT, MRI, PET-CT, and US for detecting LN metastasis in HNSCC with clinically negative neck lymph nodes. **Methods**: A systematic search was performed using Web of Science, PubMed, Scopus, Embase, and Cochrane databases. Studies comparing CT, MRI, PET-CT, or US to detect cervical metastases in HNSCC were identified. The quality of the studies was assessed using the QUADAS-2 instrument. The positive likelihood ratios (+LR) and negative likelihood ratios (−LR), sensitivity (SEN), specificity (SPE), and diagnostic odds ratio (DOR), with 95% confidence intervals (C.I.), were calculated. Analysis was stratified according to lymph node and patient basis. **Results:** Fifty-seven studies yielded 3791 patients. At the patient level, PET-CT exhibited the highest diagnostic performance, with a SEN of 74.5% (95% C.I.: 65.4–81.8%) and SPE of 83.6% (95% C.I.: 77.2–88.5%). PET-CT also demonstrated the highest +LR of 4.303 (95% C.I.: 3.082–6.008) and the lowest −LR of 0.249 (95% C.I.: 0.168–0.370), resulting in the highest DOR of 15.487 (95% C.I.: 8.973–26.730). In the evaluation of diagnostic parameters for various imaging modalities on node-based analysis results, MRI exhibited the highest SEN at 77.4%, and PET demonstrated the highest SPE at 96.6% (95% C.I.: 94.4–98%). PET-CT achieved the highest DOR at 24.353 (95% C.I.: 10.949–54.166). **Conclusions:** PET-CT outperformed other imaging modalities across the majority of studied metrics concerning LN metastasis detection in HNSCC.

## 1. Introduction

Lymph node status is a critical prognostic factor in head and neck cancers. For patients with clinically positive (cN+) neck lymph node metastasis, the standard treatment is modified radical neck dissection. In contrast, patients with a clinically negative (cN0) neck can be managed through elective neck dissection or by adopting a watchful waiting strategy. Notably, staging cervical lymph node metastasis through palpation is frequently unreliable, as occult cervical nodal metastases are present in at least 30% of cases evaluated using this technique [1]. With the advancement of modern imaging modalities, clinical staging incorporates both physical examination and the findings of other imaging modalities [2]. The current literature focuses on developing a staging method with enough sensitivity to lower the risk of occult metastases to under 20% [2].

Traditional imaging methods for head and neck squamous cell carcinoma (HNSCC), such as computed tomography (CT) and magnetic resonance imaging (MRI), have limited effectiveness in detecting occult cervical lymph node metastases [3,4]. Positron emission tomography/computed tomography (PET-CT) has emerged as a relatively novel diagnostic method, gaining popularity in assessing HNSCC and other cancers that metastasize to the cervical lymph nodes. However, despite its growing application, there is a scarcity of literature focusing on PET-CT’s role in detecting cervical lymph node metastases. Existing studies show varied outcomes and differing study populations, complicating comparative analyses [4,5,6,7].

The management of HNSCC varies significantly based on the primary tumor site. For instance, the approach to treating oropharyngeal cancer may differ from that of laryngeal or oral cavity cancers due to differences in tumor biology, patterns of metastasis, and response to treatment. Oropharyngeal cancers, particularly those associated with human papillomavirus (HPV), tend to have a better prognosis and may be managed with less aggressive treatment compared to HPV-negative tumors. Conversely, cancers of the larynx may require more aggressive surgical intervention due to their tendency to invade local structures and their impact on critical functions such as speech and breathing.

Previous meta-analyses have evaluated the diagnostic accuracy of different imaging techniques for assessing neck node involvement [2,8,9,10,11]. However, these analyses mostly included both cN+ and cN0 patients without a specific focus on those with cN0 necks. Furthermore, previous meta-analyses did not differentiate between PET alone and combined PET-CT, and some excluded PET entirely [2,8,9,10,11]. Combined PET-CT has been shown to enhance the precision of staging. Since their publication, many recent studies have presented findings that challenge their conclusions [12,13,14,15]. Our review addresses these limitations by including a larger number of studies, performing subgroup analyses to compare the diagnostic performance of different imaging modalities, and incorporating newer imaging techniques such as PET-CT.

This study aims to perform a systematic review and meta-analysis to assess the effectiveness of various imaging modalities—CT, MRI, PET, and US—in detecting neck lymph node metastasis in cN0 HNSCC patients.

## 2. Methods

Our study conformed to the Preferred Reporting Items for Systematic Reviews and Meta-analyses (PRISMA) guidelines and the recommendations delineated in the Cochrane Handbook for Systematic Reviews of Interventions [16,17].

### 2.1. Literature Search

A comprehensive literature review was carried out across several databases, including Web of Science, PubMed, Scopus, Embase, and Cochrane, without applying any date restrictions. Furthermore, a manual examination of reference lists and meta-analyses was conducted to uncover additional pertinent references. The search methodology entailed amalgamating diverse terms as outlined below: (((MRI OR “PET-CT” OR PET OR CT OR “X-ray” OR “X ray” OR ultrasound OR US OR “positron emission tomography” OR “magnetic resonance imaging” OR “computed tomography” OR “MR imaging” OR “FDG” OR “PET-CT” OR “positron emission tomography-computed tomography” OR USG OR CECT OR Ultrasonography OR “2-deoxy-2-[fluorine-18]fluoro-D-glucose” OR “^1^⁸F-FDG-PET-CT” OR “^1^⁸F-FDG-PET-MR”) AND (“Node metastases” OR “Node metastasis” OR “Lymph node” OR “LN metastasis” OR “LN metastases”)) AND (“head and neck” OR Head OR “Oral squamous cell carcinoma” OR OSCC OR “oral cancer” OR “Oral carcinoma” OR “oral tongue squamous cell carcinoma” OR OTSCC OR neck OR HNSCC OR hypopharyngeal OR oropharyngeal OR laryngeal OR mouth OR Oropharynx)). The last search in the engines was conducted on 20 April 2024.

### 2.2. Eligibility Criteria

Two independent reviewers screened references and evaluated their eligibility. Studies comparing imaging methods for HNSCC were included in the meta-analysis if they met the following criteria: (1) Written in English; (2) Included patients with biopsy-proven HNSCC and clinically negative neck lymph nodes (cN0); (3) Used histopathology from neck dissection as the reference standard; (4) Employed CT, MRI, PET, or US to detect cervical metastases; (5) Provided sufficient data to calculate true-positive or false-negative values. Exclusion criteria were (1) Lack of raw data presentation; (2) Inclusion of both cN0 and cN+ patients without separate data for cN0 patients.

### 2.3. Data Collection

A standardized methodology for data extraction was utilized, employing an offline sheet to collect relevant information from each study included. The extracted data included study characteristics and patient demographics: primary author and publication year, study location, number of active patients and controls, age groupings, tumor site, TNM staging, neck dissection, acquisition time, primary treatment, inclusion criteria, primary outcomes, and study conclusions. In addition, imaging details: modality used, criteria for positive results. Moreover, outcomes: true positive, false positive, true negative, and false negative values.

### 2.4. Quality Assessment

A systematic evaluation of the included studies’ methodological quality was performed using the QUADAS-2 instrument. This tool was chosen because it is widely accepted and provides a comprehensive evaluation of the risk of bias and applicability of diagnostic accuracy studies. QUADAS-2 assesses four key domains: patient selection, index test, reference standard, and flow and timing. Each domain is evaluated for risk of bias and concerns regarding applicability [18].

### 2.5. Data Synthesis

Data synthesis and heterogeneity analysis were performed using the open-source software OpenMeta Analyst V0.1. Effect sizes were calculated using a random-effects model, incorporating sensitivity (SEN) and specificity (SPE) measures. The presence of heterogeneity was assessed using the I^2^ statistic. Multiple metrics were employed to evaluate the diagnostic performance of the imaging modalities: positive likelihood ratios (+LR) and negative likelihood ratios (−LR), SEN, SPE, and diagnostic odds ratio (DOR), each accompanied by 95% confidence intervals (C.I.). High SEN and SPE values indicated superior screening accuracy. +LR and −LR influenced misdiagnosis rates, while DOR provided insight into the discriminatory ability of the tests. An elevated DOR value signified a stronger discriminative capacity for distinguishing between imaging modalities. Statistical heterogeneity was quantified using I-squared (I^2^) and chi-squared (X^2^) statistics, with X^2^ *p* < 0.10 and I^2^ ≥ 50% indicative of significant heterogeneity.

## 3. Results

### 3.1. Literature Search

**Figure 1:**
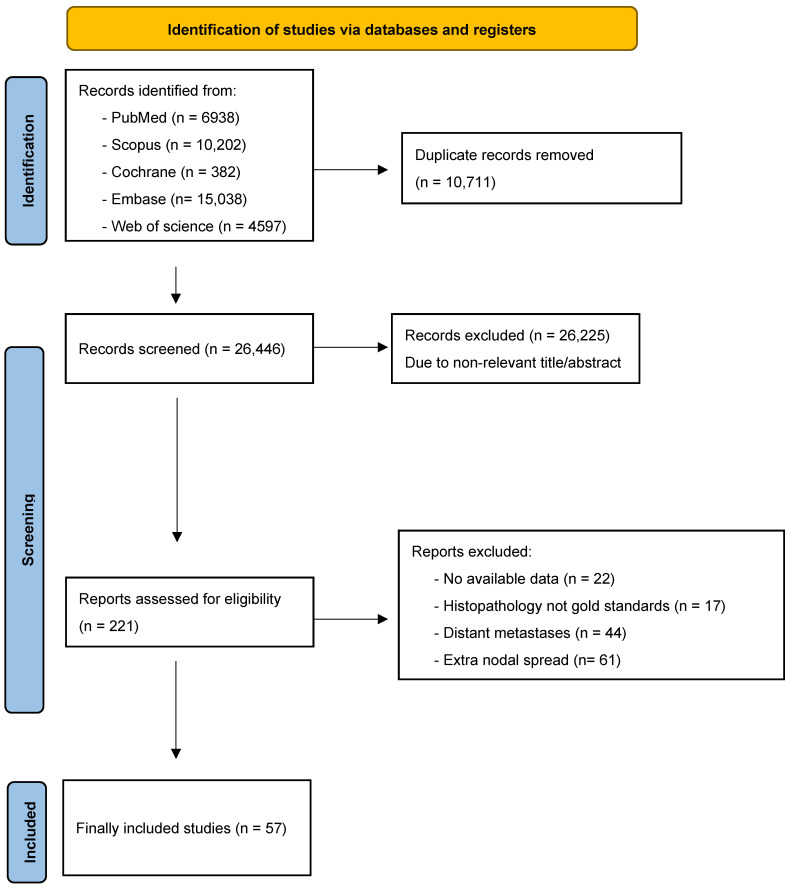
PRISMA flow chart.

A comprehensive systematic literature review was conducted, yielding an initial pool of 36,307 studies. Duplicate removal left 26,446 unique articles for consideration. The title and abstract screening process further narrowed the selection to 221 records, and subsequent full-text screenings, employing predefined exclusion criteria, resulted in the exclusion of an additional 164 studies. Ultimately, a total of 57 studies were incorporated into the final systematic review and meta-analysis [1,4,12,13,14,15,19,20,21,22,23,24,25,26,27,28,29,30,31,32,33,34,35,36,37,38,39,40,41,42,43,44,45,46,47,48,49,50,51,52,53,54,55,56,57,58,59,60,61,62,63,64,65,66,67,68,69]. The study selection process and the reason behind exclusion are shown in the PRISMA flow diagram (Figure 1).

### 3.2. Included Studies Characteristics

The present meta-analysis systematically consolidated data from a collective of 57 studies, encompassing a substantial cohort of 3791 patients. Most studies employed a rigorous methodology, with consistent documentation of TNM staging, including both clinical and pathological staging methodologies as referenced in the studies by Akoglu et al. and Bae et al., respectively [4,19]. Imaging protocols and equipment were scrutinized, noting the use of specific scanners, such as the CT 9800 HLA and Magnetom SP-10 scanners, alongside corresponding acquisition times. A predominant focus on surgical intervention as the primary treatment modality emerged, with studies like Barchetti et al. exclusively employing surgical resection [20]. However, Bae et al. introduced a combination approach, incorporating radiotherapy for a subset of their cohort (45 cases; 25.3%) [4]. A comprehensive overview of the characteristics of the included studies has been presented in Supplementary Table S1.

### 3.3. Quality Assessment

Most of the modalities used in our studies exhibited high applicability in the domains of participant selection, index test methodology, and reference standard criteria. Conversely, the bias risk assessment revealed a predominantly unclear risk of bias. A comprehensive summary of these evaluations, including detailed judgments, is presented in Supplementary Table S2.

### 3.4. Diagnostic Meta-Analysis Outcomes

#### 3.4.1. Diagnostic Detection of Lymph Node Metastasis with Node as a Unit of Analysis

**Figure 2:**
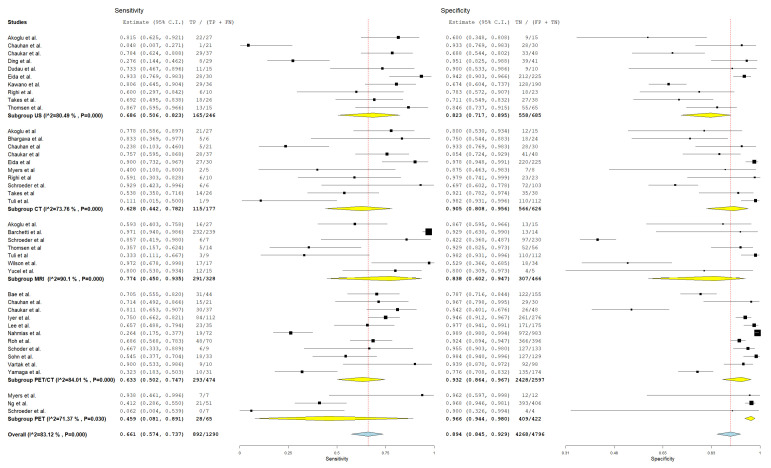
Forest plot of estimates of sensitivity and specificity for different imaging modalities in the Detection of Lymph Node Metastasis with Node as a Unit of Analysis. Included studies [4,13,19,20,26,27,28,30,31,34,35,39,41,42,46,49,52,53,54,57,58,60,61,64,66,68].

**Figure 3:**
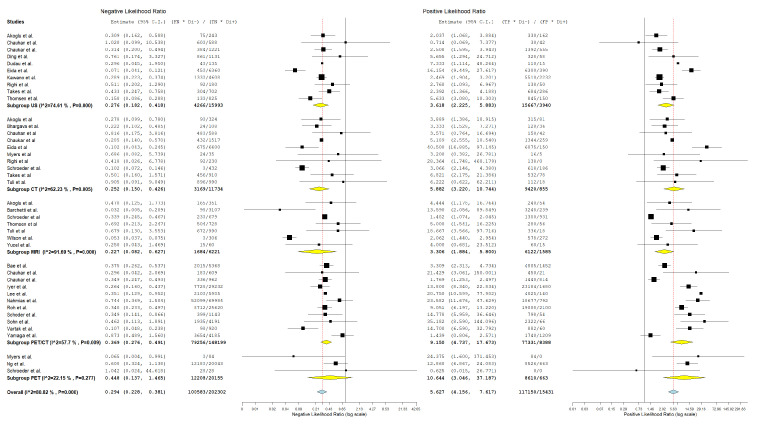
Forest plot of estimates of negative likelihood ratio and positive likelihood ratio for different imaging modalities in the Detection of Lymph Node Metastasis with Node as a Unit of Analysis. Included studies [4,13,19,20,26,27,28,30,31,34,35,39,41,42,46,49,52,53,54,57,58,60,61,64,66,68].

**Figure 4:**
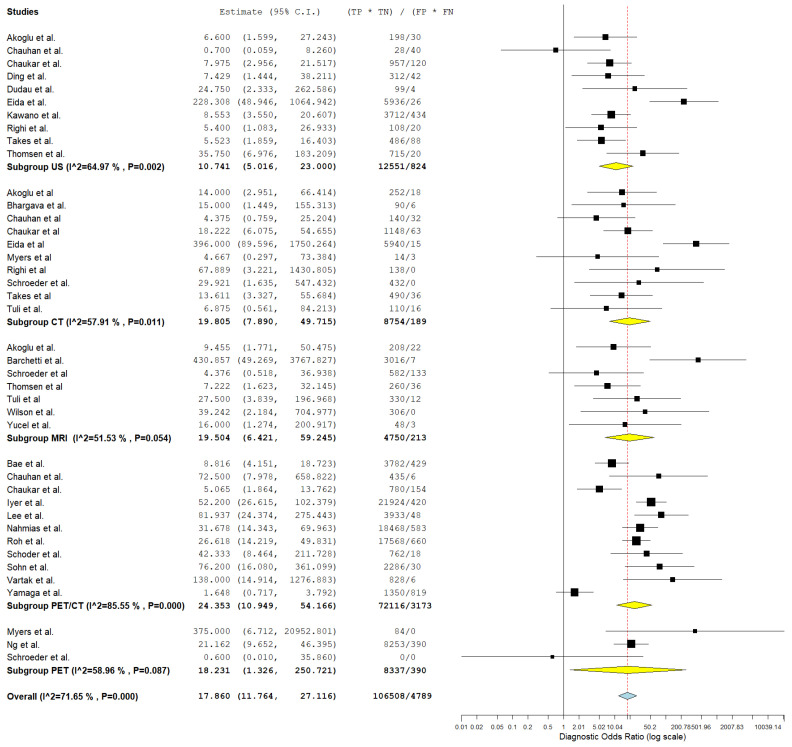
Forest plot of estimates of diagnostic odds ratio for different imaging modalities in the Detection of Lymph Node Metastasis with Node as a Unit of Analysis. Included studies [4,13,19,20,26,27,28,30,31,34,35,39,41,42,46,47,49,52,53,54,57,58,60,61,64,66,68].

In evaluating the diagnostic parameters of various imaging modalities in this subgroup, the highest SEN was observed with MRI at 77.4%. PET achieved the SPE at 96.6%. The highest DOR was found in PET-CT at 24.353. PET-CT also had the highest positive +LR at 9.150. Lastly, the lowest −LR was noted in MRI at 0.227. Most of the pooled studies in the different subgroups were heterogeneous, as evidenced by χ^2^ *p* < 0.10 and I^2^ > 50%. (Figure 2, Figure 3 and Figure 4).

##### US

Regarding US, SEN was 68.6% (95% C.I.: 50.6–82.3%), SPE was 82.3% (95% C.I.: 71.7–89.5%), DOR was 10.741 (95% C.I.: 5.016–23.000), +LR was 3.618 (95% C.I.: 2.225–5.883), and −LR was 0.276 (95% C.I.: 0.182–0.418).

##### CT

With regard CT, SEN was 62.8% (95% C.I.: 44.2–78.2%), SPE was 90.5% (95% C.I.: 80.8–95.6%), DOR was 19.805 (95% C.I.: 7.890–49.715), +LR was 5.882 (95% C.I.: 3.220–10.744), and −LR was 0.252 (95% C.I.: 0.150–0.426). (Figure 2, Figure 3 and Figure 4).

##### MRI

With regard to MRI, SEN was 77.4% (95% C.I.: 45.0–93.5%), SPE was 83.8% (95% C.I.: 60.2–94.7%), DOR was 19.504 (95% C.I.: 6.421–59.245), +LR was 3.306 (95% C.I.: 1.884–5.800), and −LR was 0.227 (95% C.I.: 0.082–0.627). (Figure 2, Figure 3 and Figure 4).

##### PET-CT

Regarding PET-CT, SEN was 63.3% (95% C.I.: 50.2–74.7%), SPE was 93.2% (95% C.I.: 86.4–96.7%), DOR was 24.353 (95% C.I.: 10.949–54.166), +LR was 9.150 (95% C.I.: 4.737–17.673), and −LR was 0.369 (95% C.I.: 0.276–0.491). (Figure 2, Figure 3 and Figure 4).

##### PET

Concerning PET, SEN was 45.9% (95% C.I.: 8.1–89.1%), SPE was 96.6% (95% C.I.: 94.4–98%), DOR was 18.231 (95% C.I.: 1.326–250.721), +LR was 10.644 (95% C.I.: 3.046–37.187), and −LR was 0.448 (95% C.I.: 0.137–1.465). (Figure 2, Figure 3 and Figure 4).

#### 3.4.2. Diagnostic Detection of Lymph Node Metastasis with Patient as a Unit of Analysis

PET-CT demonstrated the highest diagnostic performance with a SEN of 74.5% and SPE of 83.6%. It also achieved the highest +LR of 4.303 and the lowest −LR of 0.249, resulting in the highest DOR of 15.487. In comparison, US, MRI, CT, and PET had lower diagnostic parameters, with PET-CT consistently outperforming them across all measures. Most of the pooled studies in the different subgroups were heterogeneous, as evidenced by χ^2^ *p* < 0.10 and I^2^ > 50%.

##### US

**Figure 5:**
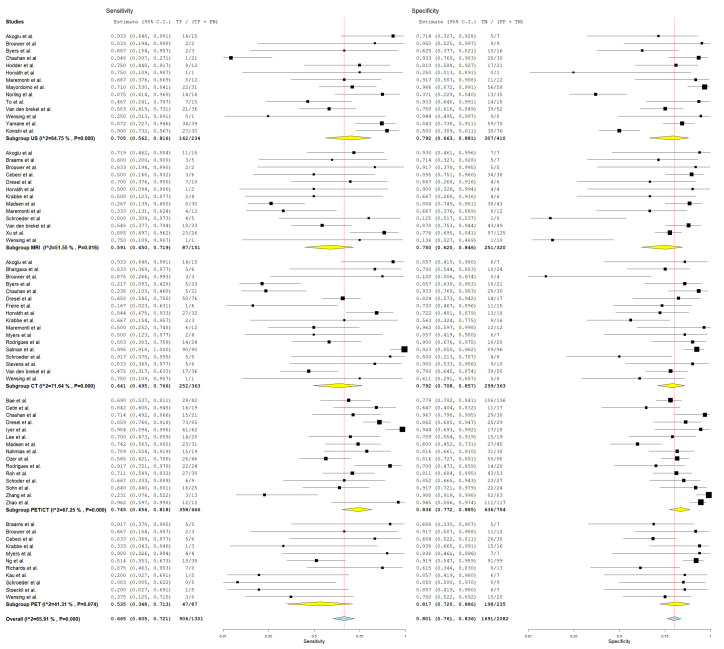
Forest plot of estimates of sensitivity and specificity for different imaging modalities in the Detection of Lymph Node Metastasis with Patient as a Unit of Analysis. Included studies [1,12,14,15,19,21,22,23,24,25,26,29,32,33,34,35,36,37,38,39,40,43,44,45,46,48,49,50,51,52,54,55,56,59,62,63,65,67,69].

**Figure 6:**
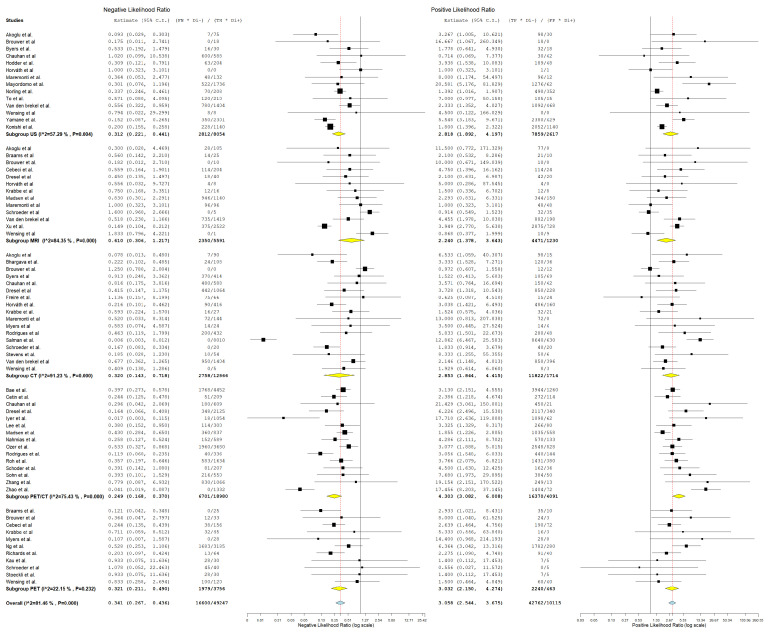
Forest plot of estimates of negative likelihood ratio and positive likelihood ratio for different imaging modalities in the Detection of Lymph Node Metastasis with Patient as a Unit of Analysis. Included studies [1,12,14,15,19,21,22,23,24,25,26,29,32,33,34,35,36,37,38,39,40,43,44,45,46,48,49,50,51,52,54,55,56,59,62,63,65,67,69].

**Figure 7:**
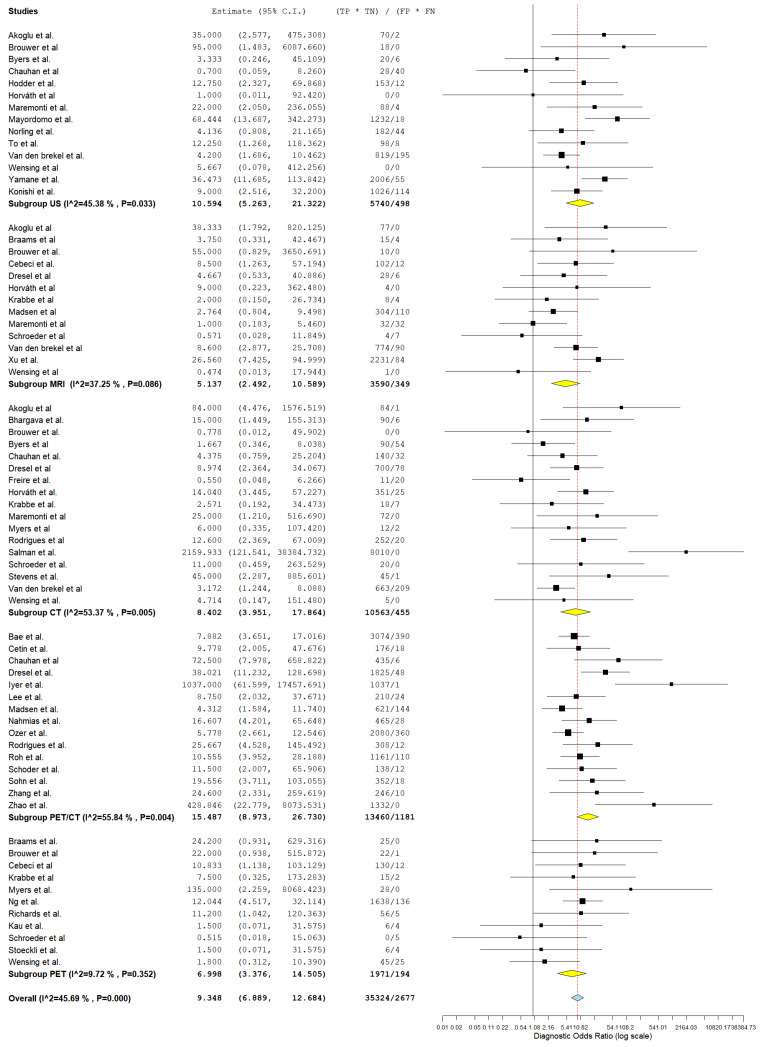
Forest plot of estimates of diagnostic odds ratio for different imaging modalities in the Detection of Lymph Node Metastasis with Patient as a Unit of Analysis. Included studies [1,12,14,15,19,21,22,23,24,25,26,29,32,33,34,35,36,37,38,39,40,43,44,45,46,48,49,50,51,52,54,55,56,59,62,63,65,67,69].

Regarding the US, the SEN was 70.5% (95% C.I.: 56.2–81.6%), and SPE was 79.2% (95% C.I.: 66.3–88.1%). The +LR for the US was 2.818 (95% C.I.: 1.892–4.197), and the −LR was 0.312 (95% C.I.: 0.221–0.441). The DOR for the US was 10.594 (95% C.I.: 5.263–21.322). (Figure 5, Figure 6 and Figure 7).

##### MRI

As regards MRI, SEN was 59.1% (95% C.I.: 45.0–71.9%), and SPE was 75.0% (95% C.I.: 62.0–84.6%). The +LR for MRI was 2.240 (95% C.I.: 1.378–3.643), and the −LR was 0.610 (95% C.I.: 0.306–1.217). The DOR for MRI was 5.137 (95% C.I.: 2.492–10.589). (Figure 5, Figure 6 and Figure 7).

##### CT

With regard to CT, SEN was 64.1% (95% C.I.: 49.5–76.6%), and SPE was 79.2% (95% C.I.: 70.8–85.7%). The +LR for CT was 2.853 (95% C.I.: 1.844–4.415), and the −LR was 0.320 (95% C.I.: 0.143–0.718). The DOR for CT was 8.402 (95% C.I.: 3.951–17.864). (Figure 5, Figure 6 and Figure 7).

##### PET-CT

Regarding PET-CT, SEN was 74.5% (95% C.I.: 65.4–81.8%), and SPE was 83.6% (95% C.I.: 77.2–88.5%). The +LR for PET-CT was 4.303 (95% C.I.: 3.082–6.008), and the −LR was 0.249 (95% C.I.: 0.168–0.370). The DOR for PET-CT was 15.487 (95% C.I.: 8.973–26.730). (Figure 5, Figure 6 and Figure 7).

##### PET

As to PET, SEN was 53.5% (95% C.I.: 34.8–71.3%), and SPE was 81.7% (95% C.I.: 72.0–88.6%). The +LR for PET was 3.032 (95% C.I.: 2.150–4.274), and the −LR was 0.321 (95% C.I.: 0.211–0.490). The DOR for PET was 6.998 (95% C.I.: 3.376–14.505). (Figure 5, Figure 6 and Figure 7).

#### 3.4.3. Diagnostic Detection of Lymph Node Metastasis with Underlying Disease HNSCC

##### US

Regarding the US, the SEN was 64.0% (95% C.I.: 46.1–75.7%), and SPE was 77.3%. The +LR for the US was 2.456, and the −LR was 0.426. The DOR for the US was 5.805. (Supplementary Figures S1–S3).

##### MRI

As regards MRI, SEN was 76.4% (95% C.I.: 52.9–90.3%), and SPE was 81.1% (95% C.I.: 66.6–90.2%). The +LR for MRI was 2.896, and the −LR was 0.250. The DOR for MRI was 15.741. (Supplementary Figures S1–S3).

##### CT

With regard to CT, SEN was 64.0% (95% C.I.: 49.7–76.2%), and SPE was 86% (95% C.I.: 80.4–90.2%). The +LR for CT was 4.513, and the −LR was 0.246. The DOR for CT was 13.257. (Supplementary Figures S1–S3).

##### PET-CT

Regarding PET-CT, SEN was 69.0% (95% C.I.: 53.4–81.3%), and SPE was 90.9% (95% C.I.: 82.1–95.6%). The +LR for PET-CT was 7.031, and the −LR was 0.336. The DOR for PET-CT was 22.532. (Supplementary Figures S1–S3).

##### PET

As to PET, SEN was 74.5% (95% C.I.: 34.8–94.1%), and SPE was 68.4% (95% C.I.: 56.1–78.5%). The +LR for PET was 2.515, and the −LR was 0.214. The DOR for PET was 8.625. (Supplementary Figures S1–S3).

#### 3.4.4. Diagnostic Detection of Lymph Node Metastasis with Underlying Disease Oral SCC

##### US

Regarding the US, the SEN was 74.7% (95% C.I.: 61.4–84.6%), and SPE was 81.5% (95% C.I.: 69.9–89.3%). The +LR for the US was 3.983, and the −LR was 0.238. The DOR for the US was 16.282. (Supplementary Figures S4–S6).

##### MRI

As regards MRI, SEN was 52.2% (95% C.I.: 31.0–72.7%), and SPE was 75.6% (95% C.I.: 53.0–89.5%). The +LR for MRI was 2.252, and the −LR was 0.589. The DOR for MRI was 4.875. (Supplementary Figures S4–S6).

##### CT

With regard to CT, SEN was 51.3% (95% C.I.: 26.0–76.1%), and SPE was 86.2% (95% C.I.: 70.2–94.3%). The +LR for CT was 3.528, and the −LR was 0.367. The DOR for CT was 8.301. (Supplementary Figures S4–S6).

##### PET-CT

Regarding PET-CT, SEN was 64.5% (95% C.I.: 50.5–76.4%), and SPE was 90.3% (95% C.I.: 81.6–95.2%). The +LR for PET-CT was 6.872, and the −LR was 0.299. The DOR for PET-CT was 19.707. (Supplementary Figures S4–S6).

##### PET

As for PET, SEN was 37.9% (95% C.I.: 20.3–59.4%), and SPE was 91.6% (95% C.I.: 77.8–97.1%). The +LR for PET was 4.299, and the −LR was 0.617. The DOR for PET was 6.768. (Supplementary Figures S4–S6).

To consolidate the findings, Table 1 provides a summary of diagnostic parameters, including sensitivity, specificity, likelihood ratios, and diagnostic odds ratios for the evaluated imaging modalities.

## 4. Discussion

The optimal management strategy for cN0 neck in SCC of the head and neck remains debated. Bayesian theory suggests that the predictive probability of neck nodal metastasis, given a negative or positive test result, depends on the pre-test probability, test SEN, and SPE [2,70]. In our diagnostic meta-analysis, encompassing 57 studies and 3791 patients, we compared different imaging modalities for detecting LN metastasis in HNSCC with cN0 neck. At the patient level, PET-CT exhibited the highest diagnostic performance, with a SEN of 74.5% and SPE of 83.6%. PET-CT also demonstrated the highest +LR of 4.303 and the lowest −LR of 0.249, resulting in the highest DOR of 15.487. In the evaluation of diagnostic parameters for various imaging modalities with Node affection as the unit of analysis, MRI exhibited the highest SEN at 77.4%. PET demonstrated the highest SPE at 96.6%. However, PET-CT achieved the highest DOR at 24.353 and the highest positive +LR at 9.150. Overall, our study showed that PET-CT consistently outperformed other imaging modalities across most studied metrics regarding LN metastasis detection in HNSCC. 

CT is pivotal in evaluating primary tumors in head and neck cancers, particularly for assessing deep tongue and mandibular involvement, as well as upstaging tumors with deep invasion into spaces like the pre-epiglottic and paraglottic regions [71]. It is instrumental in detecting nodal involvement in bone and cartilage invasion. Dual-energy and multispectral CT improve accuracy in cartilage assessment. Despite its role in complementing clinical examination in neck lymph node staging, CT has limitations in size-based assessments, potentially leading to false results and might miss microscopic nodal adenopathy [71]. MRI provides superior soft tissue delineation, aiding in tumor assessment, particularly in defining superficial tongue tumors and detecting perineural spread and intracranial extension [71]. Although it excels in evaluating cartilage invasion and perineural spread, CT outperforms MRI in detecting pathologic nodal metastases. PET and integrated PET-CT have revolutionized head and neck cancer diagnosis and staging, offering metabolic insights coupled with anatomical localization [71]. PET alone has limitations in spatial resolution, addressed by integrated PET-CT with improved localization capabilities, particularly with newer volumetric CT. PET-CT enhances TNM staging, often altering treatment plans, by accurately detecting primary tumors and regional and distant metastases, including occult lesions [71]. However, PET’s SEN might be compromised in detecting small or low metabolic lesions, especially in clinically negative necks. Despite these limitations, PET-CT significantly influences treatment decisions, highlighting its importance in head and neck cancer management for improved patient outcomes [71].

Liao et al. meta-analysis did not align with our findings as they showed that PET examination did not yield superior SEN and SPE [2]. According to their findings, CT or MRI is preferred for preoperative evaluation of cN0 neck due to their comparable diagnostic sensitivities to PET and US. Additionally, CT and MRI can assess the primary tumor status simultaneously. While the US is a cost-effective and convenient method for monitoring nodal status, it may not effectively evaluate primary tumor lesions or deep-seated lymph nodes, such as retropharyngeal nodes. The concordance between preoperative imaging and histologic specimens post-neck dissection is significant. However, the study by Liao et al. did not differentiate between PET alone and PET-CT combined, both of which showed promising results in our analysis [2]. Additionally, many studies have been conducted since the publication of their meta-analysis.

Another meta-analysis comparing CT and MRI for detecting cervical lymph node metastasis in head and neck cancer patients revealed that CT showed higher SEN (0.77) compared to MRI (0.72) when nodes were analyzed individually, while MRI exhibited higher SPE (SPE) (0.81) when neck levels were assessed [10]. MRI also had a superior area under the curve when patients were the unit of analysis. The optimal size criterion for metastatic lymph nodes was suggested as 10 mm for MRI and 12 mm for CT. Overall, MRI had higher SPE, while CT had higher SEN, indicating their complementary roles in diagnosis [11]. However, they neglected studies that incorporated PET and PET-CT studies.

Multiple studies have assessed the effectiveness of PET-CT and MRI in detecting cervical lymph node metastases in HNSCC. Linz et al. reported that PET-CT exhibited a SEN of 82.4%, SPE of 83.5%, PPV of 65.1%, and NPV of 92.7% for detecting cervical lymph node metastasis. Conversely, MRI showed a SEN of 70.6%, SPE of 62.6%, PPV of 41.4%, and NPV of 85.1%. Although the SEN and NPV in Linz et al.’s study are consistent with previous research, the SPE varied significantly, likely due to the inclusion of T3–T4 tumors [14,72]. Niu et al. reported PET-CT with a sensitivity (SEN) of 84%, specificity (SPE) of 73%, accuracy of 77%, positive predictive value (PPV) of 59%, and negative predictive value (NPV) of 91%. These values are higher than those reported in another study, likely due to their inclusion of maximum standardized uptake value and T3–T4 tumors [73]. Bae et al. found PET-CT SEN of 69.1% and SPE of 77.9%, with differences likely due to their inclusion of T3–T4 tumors [4]. Thoenissen et al. reported lower SEN (66%) and SPE (68%) with MRI in OCC patients compared to other studies, while Laimer et al. and Souren et al. reported high MRI SEN (85.7% and 83.1%, respectively) and SPE (75.6% and 75.7%), potentially influenced by T3–T4 tumor inclusion [74,75,76]. Discrepancies in these studies’ findings highlight the impact of tumor stage inclusion and the potential utility of standardized uptake value parameters in improving diagnostic algorithms for lymph node metastasis detection.

### 4.1. Perspectives and Implications

Using PET-CT for detecting malignancy in cervical lymph nodes presents challenges, primarily due to high false-positive rates and limited detection of micrometastases, as reported in the previous literature. This was attributed to inflammation increasing tissue metabolism, mimicking malignancy. PET-CT’s ability to detect micrometastases, particularly in T1–T2 HNSCC, is limited. While PET-CT can detect larger metastases effectively, its SEN decreases for smaller lesions (<5 mm). Despite advancements in technology improving image quality, PET-CT still struggles with micrometastasis detection. This limitation contrasts with sentinel node biopsy’s ability to detect even isolated tumor cells independently of glucose metabolism.

As shown in our study and the previous literature, PET-CT has consistently demonstrated efficacy in identifying patients with HNSCC; however, it may yield a high number of false positives [13,76]. Conversely, MRI is effective at identifying patients without disease but may produce a high number of false negatives. In summary, a negative PET-CT (with relatively high SEN) effectively rules out disease, while a positive MRI (with high SPE) strongly suggests disease presence. Additionally, PET-CT provides valuable information on disease spread beyond nodal staging [14,77].

In a 2017 prospective cohort study, Rohde et al. concluded that PET-CT significantly outperforms chest CT/head and neck MRI in detecting distant metastases or synchronous cancers in patients with oral cavity cancer (T1–T4) [78]. Identifying distant metastases and synchronous cancers at diagnosis significantly influences treatment decisions and prognosis. This underscores a stronger argument for upfront PET-CT utilization, not merely for enhanced SEN in detecting cervical lymph node metastases but primarily for its impact on M-classification, thus influencing treatment planning for patients with HNSCC.

Radiological assessments significantly enhance the accuracy of clinical evaluations in detecting cervical lymph node metastases in HNSCC. Clinical assessment alone, based on physical examination and palpation, often fails to detect occult metastases, leading to either overtreatment or undertreatment. The integration of imaging modalities such as CT, MRI, PET-CT, and US provides a more comprehensive evaluation, allowing for better staging and treatment planning. For instance, PET-CT, with its high sensitivity and specificity, can identify metabolic activity indicative of malignancy, which may not be apparent on clinical examination. This added value is crucial in guiding decisions for elective neck dissection or adopting a watchful waiting strategy.

Moreover, the findings of our meta-analysis have important implications for clinical practice and the management of patients with clinically negative neck lymph nodes (cN0) in HNSCC. Based on our findings, we propose the following recommendations for clinical practice and management guidelines: CT and MRI should be considered the preferred imaging modalities for preoperative evaluation of cN0 necks due to their ability to assess both the primary tumor and lymph node status. PET-CT, while effective, may not provide additional diagnostic benefits over CT and MRI and should be reserved for specific cases where metabolic information is crucial. For patients with a low pre-test probability of occult metastasis, a “watchful waiting” strategy may be justified if imaging results are negative. This approach can minimize morbidity associated with elective neck dissection. Patients with positive imaging results, even with a low pre-test probability, should undergo elective neck dissection due to the high likelihood of occult metastasis. This recommendation is based on the high positive predictive value of imaging modalities in detecting nodal metastasis.

To further validate these findings and assess their impact on clinical outcomes, we propose a prospective multicenter trial evaluating a diagnostic pathway combining CT and MRI as the first-line imaging modalities for cN0 patients. This trial should compare outcomes, such as overall survival, disease-free survival, and morbidity, between patients managed with the recommended imaging-based strategy and those managed using standard care protocols. The hypothesis is that this imaging-based strategy, tailored to pre-test probabilities and imaging results, will improve staging accuracy, optimize treatment planning, and reduce unnecessary surgical interventions, ultimately enhancing clinical outcomes for HNSCC patients with cN0 necks.

### 4.2. Strength and Limitations

To our knowledge, our meta-analysis represents the most comprehensive and up-to-date examination of all imaging modalities for detecting HNSCC. However, our meta-analysis has several limitations that should be acknowledged. First, potential publication bias is a concern, as studies with positive findings are more likely to be published than those with negative or inconclusive results. This bias could lead to an overestimation of the diagnostic accuracy of the imaging modalities evaluated. We attempted to mitigate this by conducting a comprehensive literature search across multiple databases. Second, inter-study heterogeneity is another significant limitation. The included studies varied in terms of patient populations and imaging protocol. These differences can introduce variability in the pooled estimates of sensitivity and specificity. We used a random-effects model to account for this heterogeneity, but it remains a potential source of bias. Future studies should aim for standardized imaging protocols and uniform criteria for positive results to reduce heterogeneity. Furthermore, future research should focus on stratification based on the ability to detect micro-metastasis and the precise site of HNSCC to provide more insights into diagnostic accuracy and clinical applicability.

## 5. Conclusions

Our meta-analysis assessed various imaging modalities for detecting lymph node metastasis in HNSCC, with PET-CT demonstrating superior diagnostic capabilities compared to others. Future investigations should prioritize micro-metastasis detection and focus on specific HNSCC sites to enhance diagnostic accuracy and clinical significance.

## Figures and Tables

**Table 1:** Summary of the results.

Imaging Modality	N	SEN	SPE	LR+	LR−	DOR
**Node as a Unit of Analysis**						
**CT**	803	62.8%	90.5%	5.882	0.252	19.805
**MRI**	794	77.4%	83.8%	3.306	0.227	19.504
**PET-CT**	3071	63.3%	93.2%	9.150	0.369	24.353
**PET**	487	45.9%	96.6%	10.644	0.448	18.231
**US**	931	68.6%	82.3%	3.618	0.276	10.741
**Patient as a Unit of Analysis**						
**CT**	726	64.1%	79.2%	2.853	0.320	8.402
**MRI**	471	59.1%	75.0%	2.240	0.610	5.137
**PET-CT**	1220	74.5%	83.6%	4.303	0.249	15.487
**PET**	322	53.5%	81.7%	3.032	0.321	6.998
**US**	644	70.5%	79.2%	2.818	0.312	10.594
**Underlying disease HNSCC**						
**CT**	809	64.0%	86.0%	4.513	0.246	13.257
**MRI**	506	76.4%	81.1%	2.896	0.250	15.741
**PET**	89	74.5%	68.4%	2.515	0.214	8.625
**PET/CT**	2436	69.0%	90.9%	7.031	0.336	22.532
**US**	293	64.0%	77.3%	2.456	0.426	5.805
**Underlying disease Oral SCC**						
**CT**	615	51.3%	86.2%	3.528	0.367	8.301
**MRI**	697	52.2%	75.6%	2.252	0.589	4.875
**PET**	546	37.9%	91.6%	4.299	0.617	6.768
**PET/CT**	1412	64.5%	90.3%	6.872	0.299	19.707
**US**	1153	74.7%	81.5%	3.983	0.238	16.282

## Data Availability

Data supporting this meta-analysis are derived from previously published studies, as referenced in this manuscript. Additional information may be shared upon request, subject to author permissions.

## Supplementary Materials

The following supporting information can be downloaded at https://www.mdpi.com/article/10.3390/jcm13247622/s1; Supplementary Table S1: Summary and baseline characteristics of the included studies; Supplementary Table S2: Risk of bias by QUADS-2 for the included studies. Supplementary Figure S1: Forest plot of estimates of sensitivity and specificity for different imaging modalities in the Detection of Lymph Node Metastasis in patients with underlying disease HNSCC; Supplementary Figure S2: Forest plot of estimates of negative likelihood ratio and positive likelihood ratio for different imaging modalities in the Detection of Lymph Node Metastasis in patients with underlying disease HNSCC; Supplementary Figure S3: Forest plot of estimates of diagnostic odds ratio for different imaging modalities in the Detection of Lymph Node Metastasis in patients with underlying disease HNSCC; Supplementary Figure S4: Forest plot of estimates of sensitivity and specificity for different imaging modalities in the Detection of Lymph Node Metastasis in patients with underlying disease Oral SCC; Supplementary Figure S5: Forest plot of estimates of negative likelihood ratio and positive likelihood ratio for different imaging modalities in the Detection of Lymph Node Metastasis in patients with underlying disease Oral SCC; Supplementary Figure S6: Forest plot of estimates of diagnostic odds ratio for different imaging modalities in the Detection of Lymph Node Metastasis in patients with underlying disease Oral SCC.
