# Supplementary material for: Comparing the Efficacy of CT, MRI, PET-CT, and US in the Detection of Cervical Lymph Node Metastases in Head and Neck Squamous Cell Carcinoma with Clinically Negative Neck Lymph Node: A Systematic Review and Meta-Analysis
Source: J Clin Med. 2024 Dec 14;13(24):7622. doi: 10.3390/jcm13247622 (PMC11728035; doi:10.3390/jcm13247622)
Supplement: Supplementary file 1 [file jcm-13-07622-s001.zip › Supplementary Table S1.pdf]

Table 1: Summary and baseline characteristics of the included studies

N	Study ID	Imaging method	Total sample	Site	Study design	Age, (mean±SD)y	Study period	Tumour site, n(%)	TNM Staging, n(%)	Neck dissections, n(%)	Imaging criteria	Acquisition time, min	Primary treatment, n(%)	Inclusion criteria	Conclusion
1	Akoglu et al. 2005	CT	23	Turkey	Prospective	58.65 ± 9.5	ND	HNSCC	Clinically: T1N0M0 T2N0M0	**1. Unilateral:** a. RND, 13(41.93) b. SND, 2(6.45) **2. Bilateral:** a. RND, 4(12.9) b. MRND, 8(25.81) c. SND, 4(12.91)	1. With a CT 9800 HLA scanner 2. With contrast injection 3. Continuous axial sections (5 mm) were obtained from the base of the skull to the level of the clavicles 4. G1, < 1.5cm; G2, 1.5 to 3cm and G3, >3cm 5. Diagnosed as benign or malignant upon the presence of a) CN. b) Nodal rim enhancement. c) peripheral tissue invasion.	NR	Surgical resection or (wait and see)	1. Consecutive patients with HNSCC 2. Operated at the ENT department of Dokuz Eyiul University Hospital 3. Underwent Clinical, CT, US, MRI and SPECT imaging evaluation 4. With a mean age of 58.3 years 5. Gave an informed consent	"Our data show that, despite high specificity rates, especially with SPECT, none of the currently available imaging methods are reliable evaluating the occult regional metastasis because the negative predictive values of all of these methods are rather low."
		MRI									1. With a Magnetom SP-10 scanner 2. T1, T2, Fat suppression and post-gadolinium injection T1-WE (3-5 mm) were taken in the axial and coronal planes 3. Diagnosed as benign or malignant upon the presence of a) CN. b) Nodal rim enhancement. c) peripheral tissue invasion.				
		US									1. With an RT-X400 scanner 2. With a 7.5 MHz linear array probe 3. Diagnosed as benign or malignant upon the presence of a) CN. b) peripheral tissue invasion.				
2	Bae et al. 2019	PET/CT	178	South Korea	Prospective	53 ± 10.67	Between September 2010 and August 2017	1. Tongue, 148(83.1) 2. FOM, 14(7.9) 3. Buccal, 4(2.2) 4. Hard palate, 4(2.2) 5. Lip, 4(2.2) 6. Gingiva, 3(1.7) 7. RA, 1(0.6)	Pathologically: 1. T1, 100(56.2) 2. T2, 49(27.5) 3. T3, 2(1.1) 4. T4, 27(15.2) a. N0, 136(76.4) b. N1, 26(14.6) c. N2a, 14(14.6) d. N2b, 2(1.1)	LI to LIII, 178(100)	1. Performed with 18F-FDG PET/CT scanner 2. Fasted for at least 6 hours before the imaging with serum BGL <150mg/dl 3. Diagnosed as suspicious for metastasis if LNs appeared: a) > 1cm or b) Spherical in shape, b) ≥3 LNs in the first drainage area, c) Rim enhancement, CN or ECS, or d) Upon FDG Uptake criteria	NR	1. Surgery, 119(66.9) 2. Surgery plus RT, 45(25.3) 3 Surgery plus CRT, 14(7.9)	1. Between September 2010 and August 2017 2. OSCC patients who underwent surgery at the time of enrollment 3. Received prospective imaging evaluation of the whole body before 3 weeks of primary surgery 4. With an average age of 53 years 5. Gave an informed consent	"18F-FDG PET/CT can better detect occult neck metastasis than CT/MR imaging, which may potentially impact the clinical management of OCC patients."
3	Barchetti et al. 2014	MRI	80	Italy	Prospective	34–75	From January 2009 to December 2012	HNSCC, 80(100)	NR	1. RND, 57(55.34) 2. SND, 5(4.85) 3. SOND, 41(39.81)	1. Performed with a 3T(Tesla) magnet 2. Underwent contrast injection 3. The range was from the base of the skull to the level of the clavicles 4. Slice thickness was 3 mm with a field of view 220 5. Diagnosed as suspicious for metastasis if LNs appeared: a) Oval >10 mm in diameter or b) Rounded >8mm in diameter c) Any size with CN or d) Heterogenous or indistinct borders	4.9	Surgical resection, 80(100)	1. From January 2009 to December 2012 2. Consecutive patients with biopsy-proven HNSCC and nonpalpable laterocervical lymphadenopathies 3. With an age range of 34–75 years 4. Underwent MRI as part of the routine diagnostic workup 5. Gave an informed consent	"In a DWI-negative neck for malignant lymph nodes, the planned dissection could be converted to a Wait-and-scan policy, whereas DWI-positive neck would support the decision to perform a neck dissection."

**4**	**Bhargava et al. 2015**	**CT**	30	India	Prospective	48.47 ± 13	Between December 2012 and February 2014	1. BM, 10(33.3)<br>2. Larynx, 9(30)<br>3. Tongue, 6(20)<br>4. OC, 2(6.7)<br>5. Lower alveolus, 2(6.7)<br>6. Lip, 1(3.3)	Pathologically:<br>1. T1, 3(10)<br>2. T2, 11(36.67)<br>3. T3, 15(50)<br>4. T4, 1(3.3)<br>a. N0, 6(20)<br>b. N+, 24(80)	SND, 30(30)	1. Performed with a Siemens Definition AS Plus 128 slice CT scanner<br>2. Underwent contrast injection<br>3. The range was from the base of the skull to the level of the clavicles<br>4. Spiral acquisition was done at 5 mm thickness<br>5. Diagnosed as suspicious for metastasis if LNs appeared: a) > 1cm or b) Spherical in shape or b) ≥3 LNs in the first drainage area c) Rim enhancement, CN or ECS	NR	Surgical resection with END, 30(100)	1. Between December 2012 and February 2014<br>2. Histologically proven HNSCC patients with a clinically N0 neck<br>3. The primary tumor being treated with surgery as the primary modality<br>4. With an average age of 48.47 years	"A CECT may be added to the initial assessment of a patient of HNSCC, allowing for earlier diagnosis of nodal metastasis that may allow for a better chance at survival."
**5**	**Braams et al. 1995**	**PET**	12	Netherlands	Retrospective	65.3 ± 10.4	ND	1. Tongue, 3(25)<br>2. FOM, 5(41.67)<br>3. BM, 2(16.67)<br>4. Gums, 2(16.67)	Pathologically:<br>1. T1, 2(16.67)<br>2. T2, 4(33.33)<br>3. T3, 1(8.33)<br>4. T4, 5(41.67)<br>a. N0, 6(50)<br>b. N+, 6(50)	1. MRND, 6(25)<br>2. SOND, 11(45.83)<br>3. (Wait and see), 8(33.33)	1. PET used was a Siemens ECAT 951 whole-body machine<br>2. Acquires 31 planes over an axial length of 10.8 cm<br>3. The measured resolution of the system is 6 mm full width<br>4. Small radioactive markers were placed on the tip of the chin, the mandibular angles, the mid-line of the clavicles and the jugulum<br>5. Any visually positive hot spot on each emission is considered positive	30 post-injection	Surgical resection with variable dissections, 12(00)	1. Histologically proven OSCC patients<br>2. With an average age of 66 years<br>3. Underwent PET and MRI evaluation	"Using FDG-PET, lymph node metastases squamous-cell carcinomas of the oral cavity can be visualized with a high sensitivity and specificity. FDG-PET can be an improvement in the evaluation of the neck."
		**MRI**									1. Was performed with a 1.5 Tesla Philips Gyroscan S-15<br>2. Axial and coronal slices were made with slice thickness varying between 3-5 mm<br>3. Diagnosis upon the presence of:<br>a) LNs ≥ 11mm in the SD region or ≥10mm in the other regions<br>b) ≥3 LNS of ≥10mm in the SD region or 8mm in the other regions	NR			
**6**	**Brouwer et al. 2003**	**CT**	7	Netherlands	Prospective	NR	From January 2001 to November 2002	1. OC, 9(60)<br>2. OP, 6(40)	Pathologically:<br>1. T2, 6(40)<br>2. T3, 9(60)<br>a. N0, 12(80)<br>b. N+, 3(20)	MRND, 15(100)	1. CT was performed with a 4th gen Siemens Somaton Plus scanner<br>2. Underwent contrast injection<br>3. Diagnosed as suspicious for metastasis if LNs appeared: a) > 1cm or b) Spherical in shape or b) ≥3 LNs in the first drainage area c) Rim enhancement, CN or ECS	NR	Surgical resection, 15(100)	1. From January 2001 to November 2002<br>2. Histologically proven HNSCC patients with Clinically negative necks<br>3. Planned to undergo a resection of the primary tumour<br>4. Underwent CT, US, PET and MRI<br>5. Gave an informed consent	"It is unlikely that FDG-PET is superior to CT, MRI or USgFNAC in the detection of occult lymph node metastases in head and neck cancer patients with a palpably negative neck. The histopathological method used seems to be the most important factor for the differences in sensitivity in reported FDG-PET studies. New approaches like the use of monoclonal antibodies labelled with a positron emitter may improve the results of PET in these patients."
		**US**	11								Diagnosed as suspicious for metastasis if LNs appeared:<br>a) Short axis diameter of>7 mm<br>b) A round node is more likely to be malignant<br>c) The more hypoechogenic<br>d) CN or irregular margins				

		**PET**	15								1. Was performed with a dedicated full-ring PET scanner 2. All patients fasted overnight before the PET 3. Were made from the base of the skull to clavicles 4. Any visually positive hot spot on each emission is considered positive	10			
		**MRI**	7								1. Were done on a 1.5 Tesla imaging system 2. Slice thickness varied by 7 mm, with an interslice gap of 10% 3. Diagnosed as suspicious for metastasis if LNs appeared: a) > 1cm or b) Spherical in shape or b) ≥3 LNs in the first drainage area c) enhancement, CN or ECS	NR			
**7**	**Byers et al. 1998**	**US**	91	USA	Prospective	58 ± 10	Between January 1, 1990, and December 31, 1994	Tongue, 91(100)	Pathologically: 1. Tx or T1, 13(14.28) 2. T2, 51(56.04) 3. T3-T4, 27929.67) a. N0, 58(63.74) b. N1, 18(19.78) c. N2-N3-Nx, 15(16.48)	Unilateral SOND, 91(100)	Diagnosed as suspicious for metastasis if LNs appeared: a) > 1cm or b) Spherical in shape or b) ≥3 LNs in the first drainage area c) Rim enhancement, CN or ECS	NR	Surgical resection or (Wait and see), 91(100)	1. Between January 1, 1990, and December 31, 1994 2. Histologically proven OSCC patients with Clinically negative necks 3. All were treated with a glossectomy and at least SOND 4. Underwent CT and/or US imaging evaluation	"All patients with stage T2–T4 squamous cancers of the oral tongue should have an elective dissection of the neck. Patients with T1N0 cancer who have a double DNA-aneuploid tumor, depth of muscle invasion >4 mm, or have a poorly differentiated cancer should definitely undergo elective neck dissection. Ultrasound and computed tomography are of little value in predicting which patients have positive nodes."
		**CT**													
**8**	**Cebeci et al. 2023**	**MRI**	44	Turkey	Retrospective	66 ± 16.5	Between January 2018 and August 2020	1. Larynx or HP, 19(43.2) 2. OC, 13(29.5) 3. Skin, 8(18.2) 4. SN, 2(4.5) 5. Parotid, 2(4.5)	Pathologically: 1. T1, 6(13.6) 2. T2, 11(25) 3. T3, 13(29.5) 4. T4, 12(27.3) TX 2 4.5 a. N0, 38(86.4) b. N1, 4(9.1) c. N2B, 1(2.3) d. N3 , 1(2.3)	1. Unilateral: 23(52.57) 2. Bilateral: 21(47.73)	1. With an integrated 3-T PET/MRI scanner 2. All fasted for at least 6 hours 3. Situated on the scanning table in the supine position With the HN coil used 4. A 2D PET emission scans were logged jointly with MRI sequences and the acquisition time= 3 min 5. Diagnosed as suspicious for metastasis if LNs appeared: a) > 1cm or b) Spherical in shape or b) ≥3 LNs in the first drainage area c) enhancement, CN or ECS	Three	Surgical resection, 44(100)	1. Between January 2018 and August 2020 2. Histologically proven HNSCC patients with Clinically negative necks 3. PET/MRI scans were evaluated 4. Underwent CT and/or US imaging evaluation 5. Gave an informed consent	"PET/MRI is more sensitive and has a higher NPV compared to MRI alone, while its sensitivity was found to be comparable to that of PET. In addition, with its ability to detect pathological N0 patients, PET/MRI may significantly decrease the number of unnecessary neck dissections."
		**PET**										NR			
**9**	**Cetin et al. 2013**	**PET/CT**	36	Turkey	Retrospective	55 ± 13.5	Between September 2009 and June 2010	1. Larynx or HP, 22(61.11) 2. Tongue, 6(16.67) 3. OC/OP,4(1.11)	NR	SND, 36(100)	1. Performed in the post-therapy setting 2. With a 3D PET or CT scanner 3. Underwent contrast injection 4. All fasted for at least 6 hours 5. Images displayed as coregistered axial, coronal, and sagittal slices with a slice thickness of 3.75 mm 6. Diagnosed as suspicious for metastasis if LNs appeared: a) > 1cm or b) Spherical in shape or b) ≥3 LNs in the first drainage area c) enhancement, CN or ECS	NR	Surgical resection, 36(100)	1. Between September 2009 and June 2010 2. Histologically proven OSCC patients with Clinically negative necks 3. Required and END 4. Underwent PET and/or CT imaging evaluation	"PET/CT was a valuable tool to assess nodal stage of head and neck cancers, and should be considered before surgical treatment."

10	**Chauhan et al. 2012**	**CT**	49	India	Prospective	59.45 ± 10	From January 2007 to December 2008	1. BM, 17(34.69)<br>2. Alveolus, 8(16.33)<br>3. Lip, 6(12.24)<br>4. Tongue, 5(10.2)<br>5. FOM, 5(10.2)<br>6. RA, 4(8.16)<br>7. Larynx, 2(4.08)<br>8. GBS, 1(2.04<br>9. HP, 1(2.04)	Pathologically:<br>1. T1, 14(28.57)<br>2. T2, 17(34.69)<br>3. T3 , 10(20.41)<br>4. T4, 8(16.33)	SOND or MRND, 49(100)	1. Performed with CT 9800 scanner with contrast<br>2. Parallel to the body of the mandible from the skull base to the supraclavicular fossa with a 5-mm-thick section<br>3. Diagnosed as suspicious for metastasis if LNs appeared: a) > 1cm or b) Spherical in shape or b) ≥3 LNs in the first drainage area c) enhancement, CN or ECS	0.25	Surgical resection, 49(100)	1. From January 2007 to December 2008<br>2. Histologically proven HNSCC patients with Clinically negative necks (Non palpable)<br>3. Received no prior treatment to the head or neck region<br>4. All patients were studied using USG, CECT and 18FDG PET/CT, of the neck<br>5. With an age range from 42-82 years	"In N0 neck in head and neck squamous cell carcinoma, though FDG-PET-CT is more accurate than either USG or CECT in the staging of the neck, it is not accurate enough to alter the current treatment paradigm."
		**US**									1. A high-resolution 10 MHz 8L linear probe was used for the purpose of scanning<br>2. Diagnosed as suspicious for metastasis if LNs appeared: a) Short axis diameter of>7 mm<br>b) A round node is more likely to be malignant<br>c) The more hypoechogenic<br>d) CN or irregular margins	NR			
		**PET/CT**									1. Performed with a dedicated full-ring LSO crystal PET/CT scanner with contrast<br>2. All fasted for at least 6 hours before imaging with BGL ≥160mg/dl<br>3. FDG uptake staged on a 5-point scale as follows:<br>0 = no abnormal uptake, 1 =benign, 2 =probably benign, 3 =probably malignant, and 4 = definitely malignant. Scores of 3 and 4 were considered to be positive results for tumor involvement				
11	**Chaukar et al. 2014**	**US**	70	India	Prospective	51 ± 10.5	ND	1. Gingiva, 49(70)<br>2. Tongue, 18(25.71)<br>3. Lip, 3(4.29)	Pathologically:<br>1. T1-T2, 24(34.29)<br>2. T3-34, 46(65.71)	1. SOND, 48(56.47)<br>2. MRND, 37(43.53)<br>a. Unilateral, 70(82.35)<br>b. Bilateral, 15(17.65)	1. Performed with a 5–10 MHz linear transducer on a GE Voluson 730PRO US machine<br>2. With the patient in the supine position and neck in hyperextension<br>3. Scanning was from the root of the neck to the carotid sheath anteriorly to the mastoid process posteriorly<br>4. Diagnosed as suspicious for metastasis on a) Presence or absence of a hilum or b) Heterogeneity of echo texture, c) CN or Eccentric Coagulative hyperplasia	NR	Surgical resection, 70(100)	1. Histologically proven OSCC patients with Clinically negative necks<br>2. Received no prior treatment to the head or neck region<br>3. All patients were studied using USG, CECT and 18FDG PET/CT of the neck<br>4. With an age range from 29-71 years	"The quest for the most accurate imaging modality in clinically node-negative necks continues. US alone is inadequate. While PET-CT may not be a specific imaging modality in detecting occult cervical nodal metastasis in endemic regions of chronic granulomatous diseases, the performance of PET-CECT in this setting remains to be evaluated. CECT scan, routinely used in imaging for primary disease, is fairly accurate in detecting nodal metastasis. However, in early oral cancers that are generally treated without any imaging for the primary tumor, management of the neck will largely depend on clinical judgment."
		**CT**									1. CT scanner with contrast<br>2. Scanning was from the skull base to the level of arch of the Aorta inferiorly<br>3. Diagnosed as suspicious for metastasis if LNs appeared:<br>a) > 1cm or b) Spherical in shape or b) ≥3 LNs in the first drainage area c) Rim enhancement, CN or ECS				

		**PET/CT**									1. Performed on a Discovery ST PET-CT system with 16 16-slice CT scanner with a dedicated PET with contrast<br>2. Scanning was from the skull base to the mid-thigh level using multislice<br>3. Diagnosed based on SUC where: Nodes with SUV > 2.5 were considered as malignant.				
**12**	**Ding et al. 2021**	**US**	42	China	Retrospective	54.57 ± 14.41	From January 2015 to June 2018	1. BM, 5(11.9)<br>2. Tongue, 29(69.05)<br>3. Gingiva, 4(9.52)<br>4. Lips, 1(2.38)<br>5. Palate, 1(2.38)<br>6. Mouth floor, 1(2.38)	Clinically (T1 or T2), 42(100)	NR	1. Performed using an ultrasound system<br>2. Equipped with high-frequency linear array probes with frequency of 7–12 MHz<br>3. Diagnosed as suspicious for metastasis if LNs appeared:<br>a) Rounded shape with increased AP diameter or<br>b) Loss of hilum<br>c) Blurred margins and presence of microcalcification	NR	NR	1. From January 2015 to June 2018<br>2. Histologically proven OSCC patients with Clinically negative necks<br>3. Received no prior treatment to the head or neck region<br>4. All patients were studied using conventional US or CEUS<br>5. With an age range from 23-82 years<br>6. Gave an informed consent	"Compared with conventional ultrasound, CEUS may have higher clinical value for predicting early lymph node metastasis in clinically node-negative oral cancer patients. And quantitative parameters obtained from CEUS may provide valuable information in the diagnosis of cervical lymph node metastasis."
**13**	**Dresel et al. 2003**	**PET/CT**	68	Germany	Retrospective	27–88	ND	1. Lips, 2(2.94)<br>2. Tongue, 10(14.71)<br>3. OC, 44(64.71)<br>4. OP, 7(10.29)<br>5. SGs, 3(4.41)<br>6. PNSs, 2(2.94)	Pathologically:<br>1. T1, 28(41.18)<br>2. T2, 18(26.47)<br>3. T3, 6(8.82)<br>4. T4, 16(23.53)<br>a. N0, 23(33.82)<br>b. N+, 45(66.18)	NR	1. All patients fasted for at least 6 h with BGL ≤ 120mg/dl<br>2. Performed using a Marconi Axis γ-PET2 AZ operated in list mode<br>3. Scanning started from the base of the skull<br>4. Diagnosed as suspicious for metastasis Upon FDG Uptake criteria	40	1. Only surgery, 41(60.29)<br>2. Surgery and RT/CRT, 20(29.41)<br>3. Only RT, 5(7.35)<br>4. None, 2(2.94)	1. Histologically proven HNSCC patients with Clinically negative necks<br>3. Underwent Excision or biopsy before PET imaging<br>4. All patients were studied using PET/CT, CT or MRI<br>5. With an age range from 27-88 years	"It is concluded that [18F] FDG PET with hybrid PET scanners is superior to CT and MRI in the diagnosis of primary or recurrent lesions as well as in the assessment of lymph node involvement, whereas it is inferior to ultrasound in the detection of cervical lymph node metastasis."
		**MRI**									1. Spiral CT scan or MRI of HN with contrast<br>2. Diagnosed as suspicious for metastasis if LNs appeared: a) > 1cm or b) Spherical in shape or b) ≥3 LNs in the first drainage area<br>c) Rim enhancement, CN or ECS	NR			
		**CT**													
**14**	**Dudau et al. 2014**	**US**	17	UK	Retrospective	58.2 ± 11	NR	HN, 17(100)	Pathologically (T2-T4)	RND (Unilateral or bilateral), 17(100)	1. The scanner used was an Aplio XG with a 12L5 linear array<br>2. Performed as B-Mode US or CEUS<br>3. Diagnosed as suspicious for metastasis if LNs appeared:<br>a) > 1cm or b) Spherical in shape or b) ≥3 LNs in the first drainage area<br>c) Rim enhancement, CN or ECS	NR	NR	1. Histologically proven HNSCC patients with Clinically negative necks<br>2. These patients had stage T2 to T4 N0 tumors of the oral cavity, pharynx and larynx<br>3. Scheduled for surgery or LN clearance<br>4. All patients were studied using US or CEUS<br>5. With an average age of 58.2 years<br>6. Gave an informed consent	"Contrast-enhanced ultrasound holds promise in the detection and characterization of metastatic nodes that would not be diagnosed as abnormal on the basis of conventional ultrasound criteria."

15	**Eida et al. 2003**	**CT**	17	Japan	Retrospective	NR	ND	FOM, Tongue, Buccal mucosa, Palate and Gingiva	ND	NR	1. Underwent scanning with a HiSpeed Advantage SG CT system 2. Scanning was performed with a collimation of 3 mm, a pitch of 1:1, a 512*512 matrix, a display field view of 23 cm 3. Diagnosed as suspicious for metastasis if LNs appeared: a) >1cm or b) Spherical in shape or b) ≥3 LNs in the first drainage area c) Rim enhancement, CN or ECS	NR	If metastasis, Neck dissections with RT and CRT	Histologically proven OSCC patients with Clinically negative necks	"A combination of contrast-enhanced helical CT and Doppler sonography is useful for the follow-up study of clinical N0 stage neck disease."
		**US**									1. Performed by using a Logiq 700 unit 2. Equipped with a wide bandwidth (range, 6 –13 MHz) transducer 3. Diagnosed as suspicious for metastasis if LNs showed: a) CN or b) No hilar echo or hilar blood flow				
16	**Freire et al. 2003**	**CT**	48	Brazil	Prospective	55.7 ± 7.5	Between March 1994 and December 1997	OC and OP, 48(100)	Pathologically: 1. T1-T2, 13(27.08) 2. T3, 14(29.17) 3. T4, 21(43.75) a. N0, 21(43.75) b. N+, 27(56.25)	END: 1. Unilateral, 32(67) 2. Bilateral, 16(33)	1. Performed with the help of a conventional CT/PACE (GE) tomograph 2. With axial sections of 5 mm (5 mm thick and 5 mm interval) 3. Diagnosed as suspicious for metastasis if LNs appeared: a) >1cm or b) Spherical in shape or b) ≥3 LNs in the first drainage area c) Rim enhancement, CN or ECS	NR	Surgical resection, 48(100)	1. Between March 1994 and December 1997 2. Histopathologically proven biopsy of OSCC patients with clinically negative necks 3. Those larger than 1.0 cm at other levels of the neck were considered to be metastases or CN 4. All patients were studied using CT 5. With an average age of 55.7 years	"Clinical examination was more efficient than CT to identify lymph node metastases. Lymphatic drainage of the head and neck regions is complex, and lymphoscintigraphy can be useful in oral and oropharyngeal carcinomas in clinical stages I and II, but further studies are necessary to standardize the methodology."
17	**Hodder et al. 2000**	**US**	49	UK	Prospective	67 ± 9.25	From August 1993–August 1995	1. FOM, 9(18.37) 2. Tongue, 21(42.86) 3. Mandibular, 5(10.2) 4. BM, 10(20.4)	ND	SND, 35(70)	1. Carried out the ultrasound examination using an Aloka 2200 machine with a 7.5 MHz probe 2. Carried out with a 21-G needle and a minimum of three passes 3. Diagnosed as suspicious for metastasis if LNs appeared: a) Short axis diameter of>8 mm b) A round node is more likely to be malignant c) The more hypoechogenic d) CN or irregular margins	NR	Surgical resection and/or Radiotherapy to those have ≥ 3 LNs, 49(100)	1. From August 1993–August 1995 2. Histopathologically proven biopsy of OSCC patients with clinically negative necks 3. Those larger than 1.0 cm at other levels of the neck were considered to be metastases or CN 4. Scheduled for surgery or LN clearance 5. All patients were studied using US or USFNAC 6. With an average age of 67 years	"We find that US, with or without FNAC, is an accurate (86%), sensitive (92%) and specific (83%) technique for the preoperative assessment of lymph node metastases in patients with SCC."
18	**Horváth et al. 2020**	**CT**	62	Hungary	Retrospective	67 ± 8.5	Between September 2012 and August 2017	1. OC, 2(3.2) 2. OP, 6(9.7) 3. HP, 20(32.3) 4. Supraglottic, 14(22.6) 5. Glottic, 6(9.7) 6. TG, 13(21) 7. SG ,1(1.6)	NR	SND: 1. Ipsilateral, 50(80.65) 2. Bilateral, 12(19.35)	Considered abnormal by the radiologist if they were larger than 10 mm in their shortest diameter, contained central necrosis, or had a round shape	NR	At least surgical resection, 62(100)	1. Between September 2012 and August 2017 2. Histopathologically proven biopsy of HNSCC patients with clinically negative necks 3. Scheduled for surgery or LN clearance 4. All patients were studied using US, CT and MRI 5. With an average age of 67 years	"Neither the sensitivity of imaging modalities (CT, MRI, US) nor FNAC reached 100%, none of these methods can definitively exclude the presence of regional tumor metastasis. According to these data, no permissive alteration should be allowed from the current guidelines (e.g. NCCN) based on imaging/FNAC examinations
		**US**													

		**MRI**													regarding the need for elective neck dissection."
19	**Iyer et al. 2010**	**PET/CT**	111	Australia	Retrospective	NR	Between May 2003 and November 2007	HN, 80(100)	NR	1. RND, 6(4.23)<br>2. MRND, 31(21.831)<br>3. SND, 105(73.939)	1. PET/CT scans were performed using standardized techniques with IV 18F-FDG at a dose of 120 to 440 MBq as a contrast<br>2. Fasted for at least 6 hours before imaging<br>3. Scanning began from the skull vertex to at least the lower abdomen<br>4. Diagnosed as suspicious for metastasis Upon FDG Uptake criteria	ND	Surgical resection, 80(100)	1. Between May 2003 and November 2007<br>2. Histopathologically proven biopsy of HNSCC patients<br>3. Scheduled for surgery or LN clearance<br>4. All patients were studied using PET/CT<br>5. Gave an informed consent	"Despite advances in PET/CT technology, these statistics suggest that PET/CT is not sufficiently accurate to direct super selective neck dissections or to guide therapeutic decisions in the N0 neck."
20	**Kau et al. 1999**	**PET**	70	Germany	Prospective	29-78	ND	1. Larynx, 16(22.8)<br>2. OP, 37(52.8)<br>3. HP, 13(18.6)<br>4. Lips, 1(1.4)<br>5. Auricles, 1(1.4)<br>6. Unknown, 2(2.8)	Pathologically:<br>1. T1, 17(24.3)<br>2. T2, 25(35.7)<br>3. T3, 12(17.2)<br>4. T4, 16(22.8)<br>a. N0, 35(50)<br>b. N1, 7(10)<br>c. N2a, 7(10)<br>d, N2b, 16(22.8)<br>e, N2c, 4(5.7)<br>f, N3, 1(1.4)	1. Ipsilateral RND, 34(48.6)<br>2. Ipsilateral RND and contralateral functional ND, 36(51.4)	1. For PET imaging, a scanner(model ECAT EXACT or ECAT 951/R; Siemens/CTI, Munich, Germany) was used with contrast<br>2. Fasted for at least 4 hours before imaging<br>3. A 16.2- or a 10.8-cm axial field of view and yields 31 or 47 image planes per bed position<br>4. Diagnosed as suspicious for metastasis Upon FDG Uptake criteria	20	surgery alone, surgery and radiotherapy, combined radiotherapy and chemotherapy, or primary radiotherapy and type of neck dissection was decided by an interdisciplinary conference	1. Consecutive patients of HNSCC confirmed histopathologically<br>2. With an age range from 29 to 78 years<br>3. All patients were studied using PET, CT and MRI<br>4. Gave an informed consent	"A short PET protocol that is suitable for routine clinical use is superior to morphologic procedures (computed tomography and magnetic resonance imaging) for the detection of lymph node involvement in head and neck squamous cell carcinomas."
		**CT**									Diagnosed as suspicious for metastasis if LNs appeared: a) > 1cm or b) Spherical in shape or b) ≥3 LNs in the first drainage area<br>c) Rim enhancement, CN or ECS	NR			
		**MRI**										NR			
21	**Kawano et al. 2022**	**US**	123	Japan	Retrospective	NR	Between 2004 and 2016	Tongue, 123(100)	Pathologically:<br>11. T1, 97(78.9)<br>2. T2, 23(18.7)<br>3. T3, 3(2.4)<br>a. N0, 95(77.2)<br>b. N+, 28(22.8)	SND or (Wait and see), 123(100)	1. Performed using a 13-MHz linear probe (Sequoia 512, Mochida Siemens Medical Systems)<br>2. Considered abnormal by the radiologist if they were ≥10mm in their shortest diameter, contained central necrosis, or had a round shape	NR	NR	1. Between 2004 and 2016<br>2. Patients with clinical T1–2N0 tongue SCC<br>3. Upon initial assessment, who underwent an intraoral US examination	"These results suggest that intraoral US findings closely reflect pathological findings and could be useful to predict occult nodal metastasis in patients with early-stage tongue cancer."

**22**	**Konishi et al. 2022**	**US**	106	Japan	Retrospective	62.2 ± 16.8	Between September 2014 and September 2020	Tongue, 106(100)	Clinically:<br>1. T1, 41(38.68)<br>2. T2, 65(61.32)	NR	1. Performed using the ProSound alpha 7 system (Hitachi-Aloka Medical, Japan) using a 7.5 MHz/38 or 50 mm linear probe<br>2. The acoustic coupling material of 10mm thickness<br>3. Considered abnormal by the radiologist if they were ≥10mm in their shortest diameter, contained central necrosis, or had a round shape	NR	1. Surgical resection, 77(72.64)<br>2. RT, 29(27.34)	1. Between September 2014 and September 2020<br>2. One hundred and twenty-four patients who had primary tongue cancer<br>3. All patients were studied using US Imaging<br>4. With an average age of 62.2 years<br>5. Gave an informed consent	"Irregular margins and internal echo intensity of the tongue cancer lesion assessed using IUS may predict the occurrence of late cervical lymph node metastasis in T1/2N0M0 cases."
**23**	**Krabbe et al. 2008**	**CT**<br>**MRI**<br>**PET**	38	Netherlands	Retrospective	60 ± 11.556	From December 1999 till December 2003	1. OC, 16(42.1)<br>2. Gums, 7(18.42)<br>3. FOM, 13(18.6)<br>4. Tongue base, 2(5.26)<br>5. Tonsillar fossa, 3(7.89)	Pathologically:<br>1. T1, 15(39.47)<br>2. T2, 11(28.95)<br>3. T3, 5(13.16)<br>4. T4, 7(18.42)	END, 33(86.84)	Diagnosed as suspicious for metastasis if LNs appeared: a) > 1cm or b) Spherical in shape or b) ≥3 LNs in the first drainage area c) Rim enhancement, CN or ECS<br>1. Scans acquired via A Siemens CTI ECAT 951 (31 planes over 11.8 cm) and a Siemens CTI ECAT EXACT HR + (63 planes over 15.5 cm)<br>2. 6mm full width at half maximum transaxially in the center of the field of view , and 5 mm<br>3. Fasted for at least 4h before Contrast injection<br>4. Diagnosed as suspicious for metastasis Upon FDG Uptake criteria	NR	Surgical resection and/or biopsy	1. From December 1999 till December 2003<br>2. All patients with a newly diagnosed SCC of the oral cavity or oropharynx<br>3. All patients were studied using US Imaging<br>4. With an average age of 60 years	"Although FDG PET performed better than conventional imaging modalities, sensitivity was lower than desired. As a consequence, clinical application of FDG PET in the patient staged as N0 is limited."
**24**	**Lee et al. 2015**	**PET/CT**	39	South Korea	Retrospective	65 ± 10.75	From July 2006 to June 2013	1. Pyriform sinus, 27(69.23)<br>2. Postcricoid region, 3(7.69)<br>3. Posterior wall, 9(23.1)	Pathologically:<br>1. T1, 7(17.95)<br>2. T2, 20(51.28)<br>3. T3, 8(20.51)<br>4. T4, 4(10.26)<br>a. N0, 19(48.72)<br>b, N1, 10(25.64)<br>c, N2b, 5(12.82)<br>d, N2c, 5(12.82)	MRND or SND, 39(100)	1. Formed on one of two CT scanners (Somatom Sensation 16 or 64; Siemens, Erlangen, Germany) with contrast<br>2. Contiguous 3-mm scans of the neck were acquired in the axial plane<br>3. From the skull base to the carina<br>4. CT diagnosed as suspicious for metastasis if LNs appeared: a) > 11mm or b) Spherical in shape or b) ≥3 LNs in the first drainage area<br>c) Rim enhancement, CN or ECS<br>5. PET diagnosed as suspicious for metastasis Upon FDG Uptake criteria	Three	Surgical resection, 39(100)	1. From July 2006 to June 2013<br>2. Histopathologically proven biopsy patients of HNSCC with cN0<br>3. All patients were studied using PET/CT Imaging<br>4. With an average age of 62.13 years<br>5. Gave an informed consent	"The addition of PET-CT examination to anatomic imaging involving CT and MR did not provide additional benefit for the preoperative evaluation of cervical nodal metastasis in patients with hypopharyngeal SCC with nonpalpable neck, yielding insufficient data to spare elective neck dissection."
**25**	**Madsen et al. 2023**	**PET/CT**	76	Denmark	Prospective	63 ± 10.76	From September 2013 to February 2016	1. FOM, 27(36)<br>2. Tongue,34(45)<br>3. UA, 1(1)<br>4. LA, 7(9)<br>5. BM, 5(7)<br>6. HP, 1(1)<br>7. RA, 1(1)	Pathologically:<br>1. T1, 53(70)<br>2. T2, 23(30)	END, 21(27.63)	1. MRI was performed on Philips Achieva, Achieva dStream or Ingenia 1.5T with contrast<br>2. Axial or coronal planes with coverage from skull base to aortic arch using 5 mm slices<br>3. Images were read on a GE Centricity RA1000 PACS workstation<br>4. Had a slice thickness of 3.27 mm<br>5. Diagnosed as suspicious for metastasis if LNs appeared:<br>a) > 1cm or b) Spherical in shape or b) ≥3 LNs in the first drainage area c) Rim enhancement, CN or ECS<br>d) FDG Uptake	2.5	Surgical excision or wait and see	1. From September 2013 to February 2016<br>2. All patients with suspicion of oral cavity cancer confirmed histopathologically (T1-T2)<br>3. All patients were studied using PET/CT or MRI<br>4. With an average age of 63 years<br>5. Gave an informed consent	"The accuracy of PET/CT and neck MRI was comparable (66% vs 63%, p = 0.85), the PPV was slightly in favor of neck MRI (56% vs 62%, p = 0.73), the NPV was slightly in favor of PET/CT (77% vs 63%, p = 0.16). Neither PET/CT nor neck MRI should stand alone for N-staging T1–T2 oral cavity cancer."

		**MRI**									1. MRI was performed on Philips Achieva, Achieva dStream or Ingenia 1.5T with contrast<br>2. Axial or coronal planes with coverage from skull base to aortic arch using 5 mm slices<br>3. Images were read on a GE Centricity RA1000 PACS workstation<br>4. Diagnosed as suspicious for metastasis if LNs appeared: a) >1cm or b) Spherical in shape or b) ≥3 LNs in the first drainage area c) Rim enhancement, CN or ECS	NR			
**26**	**Maremonti et al. 1997**	**US**	45	Italy	Retrospective	24-72	Between January 1993 and December 1995	OC, 45(100)	Pathologically:<br>1. T1, 5(11.11)<br>2. T2, 20(44.44)<br>3. T3, 14(31.11)<br>4. T4, 6(13.13)	NR	1. Performed with a 7.5MHz sonda in transverse, longitudinal and oblique planes<br>2. Diagnosed up to the presence of: a) Nodal grouping or, b) LN enlargement, or c) Alteration of nodal vascular pattern	NR	NR	1. Between January 1993 and December 1995<br>2. Consecutive patients with suspicion of oral cavity cancer confirmed histopathologically<br>3. All patients were studied using CT, US and/or MRI<br>4. With an age range from 24-72 years<br>5. Gave an informed consent	"Therefore, a diagnostic preoperative study must include echo-colour-Doppler preferably associated with computed tomography to achieve the greatest diagnostic accuracy."
		**MRI**									1. Performed with a General Electric 0.5 Tesla device<br>2. Using T1, T2 and proton density sequences in multiplanar acquisitions after injection of paramagnetic media<br>3. Diagnosed by the presence of central nodal necrosis				
		**CT**									1. Performed with a Philips Tomoscan with contrast<br>2. Images were obtained with multiple transaxial slices 3 mm thick, every 3 mm<br>3. Images were read on a GE Centricity RA1000 PACS workstation<br>4. Diagnosed as suspicious for metastasis if LNs appeared: a) > 1cm or b) Spherical in shape or b) ≥3 LNs in the first drainage area<br>c) Rim enhancement, CN or ECS				
**27**	**Mayordomo et al. 2016**	**US**	90	Spain	Prospective	60 ± 12.46	Between 2005 and 2012	1. Tongue, 45(50)<br>2. FOM, 16 (17.8)<br>3. RA, 13(14.4)<br>4. Gum, 8(8.9)<br>5. BM, 4(4.4)<br>6. Lips, 3(3.3)	Pathologically:<br>1. T1, 27(30)<br>2. T2, 35(38.9)<br>3. T3, 28(31.1)	END or (Wait and see), 90(100)	1. Philips ATL HDI 4000 (Philips Ultrasound, Bothell, WA, USA) ultrasound system version<br>2. With a 6- to 12-MHZ linear transducer and contrast<br>3. Scanning was performed from level I to V on the homolateral neck to the tumor, except if it was in the midline<br>4. Considered abnormal by the radiologist if they were ≥10mm in their shortest diameter, contained central necrosis, or had a round shape	NR	1. Excision and END, 66(73.3)<br>2. Isolated excision, 24(26.7)	1. Between 2005 and 2012<br>2. Primary OCSCC newly diagnosed<br>3. Safety margin tumoral excision with or without END<br>4. Underwent US imaging evaluation<br>5. With an average age of 60 years<br>6. Gave an informed consent	"By using selected sonographic criteria, ultrasound can be a valid preoperative diagnostic method to optimize staging cervical metastasis and to help decide about neck dissection."
**28**	**Myers et al. 1998**	**PET**	11	USA	Retrospective	59 ± 8.68	From October 1994 to May 1997	OC, 11(100)	NR	Neck dissection , 19(100)	1. PET Positioned in the ECAT 951/31R Tomograph siemens with contrast (18F FDG)<br>2. Scanning included Head, Neck and Thorax<br>3. Diagnosed as suspicious for metastasis Upon FDG Uptake criteria	NR	Surgical excision , 11(100)	1. From October 1994 to May 1997<br>2. Consecutive patients with cN0 OSCC confirmed histopathologically<br>3. Underwent PET or CT imaging evaluation with 18F FDG Contrast<br>4. With an average age of 60 years<br>5. Gave an informed consent	"PET appears to be a diagnostic aid that may be applied when evaluating N0 Neck for patients with OCC of the oral cavity.'

		**CT**									Diagnosed as suspicious for metastasis if LNs appeared: a) > 1cm or b) Spherical in shape or b) ≥3 LNs in the first drainage area<br>c) Rim enhancement, CN or ECS				
**29**	**Nahmias et al. 2007**	**PET/CT**	70	USA	Retrospective	62 ± 15	ND	HN, 70(100)	NR	SND(N0) or MRND(N+), 70(100)	1. Performed on a biograph-16 PET/CT scanner<br>2. Fasted for at least 6 hours before the imaging<br>3. Diagnosed as suspicious for metastasis if LNs appeared: a) > 1cm or b) Spherical in shape or b) ≥3 LNs in the first drainage area<br>c) Rim enhancement, CN or ECS<br>d) FDG Uptake criteria	Three to Four	Surgical resection	1. Consecutive patients with cN0 OSCC confirmed histopathologically<br>2. Underwent PET/CT imaging evaluation with 18F FDG Contrast<br>3. With an average age of 62 years<br>4. Gave an informed consent	"In patients with clinically negative necks, a negative test would not help the surgeon in the management strategy of the patient because of the rate of false-negative results, but a positive test can diagnose metastatic deposits with a high positive predictive value."
**30**	**Ng 2006**	**PET**	134	Taiwan	Prospective	52.1 ± 9.33	From January 2003 to December 2005	1. Tongue, 51(38.1)<br>2. BM, 50(37.3)<br>3. Gums, 24(17.9)<br>4. RA, 4(3)<br>5. FOM, 2(1.5)<br>6. HP, 1(0.7)	Pathologically:<br>1. T1, 30(22.39)<br>2. T2, 65(48.51)<br>3. T3, 15(11.19)<br>4. T4, 24(17.91)	SOND((levels I, II, and III), Extended SOND(levels I to IV) or MRND (levels I to V)	1. Performed on a biograph-16 PET/CT scanner<br>2. Fasted for at least 6 hours before the imaging<br>3. Diagnosed as suspicious for metastasis if LNs appeared: a) > 1cm or b) Spherical in shape or b) ≥3 LNs in the first drainage area<br>c) Rim enhancement, CN or ECS<br>d) FDG Uptake criteria	40 post-injection	None	1. From January 2003 to December 2005<br>2. Patients with cN0 OSCC confirmed histopathologically<br>3. Underwent PET imaging evaluation with 18F FDG Contrast<br>4. With an average age of 52.1 years<br>5. Gave an informed consent	"[18F]FDG PET was superior to CT/MRI for detecting palpably occult neck metastasis of oral SCC. Because [18F]FDG PET could reduce the probability of occult neck metastasis to less than 15% in T1 to T3 tumors, it should be indicated for evaluation of these subpopulations."
**31**	**Norling et al. 2014**	**US**	51	Denmark	Prospective	64.3 ± 15.25	From 1st of April 2010 to 1st of July 2012	Oral, 51(100)	Clinically:<br>1. T1, 27(52.94)<br>2. T2, 18(35.29)<br>3. T3, 2(3.92)<br>4. T4, 4(7.84)	Biopsy or SND, 51(100)	1. Performed on a GE logiq 9 using a GE 10L linear array transducer<br>2. Fasted for at least 6 hours before the imaging<br>3. Diagnosed as suspicious for metastasis if LNs appeared:<br>a) Short axis diameter of>8 mm<br>b) A round node is more likely to be malignant<br>c) The more hypoechogenic<br>d) CN or irregular margins	NR	Surgical resection, 51(100)	1. From 1st of April 2010 to 1st of July 2012<br>2. Patients with suspicion of or histopathologically verified OSCC<br>3. Only patients staged as cN0 and planned for surgical treatment of the neck<br>4. All patients underwent US Imaging evaluation<br>5. With an average age of 64.3 years<br>6. Gave an informed consent	"The addition of US to the clinical work-up of patients with cN0 OSCC increases the detection of metastases, thus, the US potentially reduces the number of patients requiring a secondary neck surgery after sentinel node biopsy."
**32**	**Ozer et al. 2012**	**PET/CT**	223	Turkey	Retrospective	NR	Between January 1, 2005, and December 12, 2007	1. OC, 89(36.6)<br>2. OP, 82(33.7)<br>3. Larynx,45(18.5)<br>4. Unknown, 18(7.4)<br>5. HP, 9(3.7)	NR	END, 169(100)	1. Performed with a Siemens Biograph 16 Hi-Res PET/CT scanner<br>2. Scanning begins at the top of the head and continues to mid-thigh<br>3. A mobilizer is then used to ensure proper alignment of the PET and CT images<br>4. Diagnosed as suspicious for metastasis if LNs appeared: a) > 1cm or b) Spherical in shape or b) ≥3 LNs in the first drainage area<br>c) Rim enhancement, CN or ECS<br>d) FDG Uptake criteria	23	Surgical resection, 169(100)	1. Between January 1, 2005 and December 12, 2007<br>2. PET/CT scans were performed for patients with cancers of the head and neck with cN0<br>3. All patients underwent PET/CT Imaging evaluation with 18F-FDG Contrast	"PET/CT has a much-reduced rate of efficacy for the clinically negative neck compared to the clinically positive neck. PET/CT in its current stage does not appear to over an advantage in staging the clinically N0 neck due to high rates of false positives and negatives."

**33**	**Richards et al. 2007**	**PET**	21	UK	Retrospective	NR	ND	Oral, 21(100)	Pathologically: (T1-T2), 21(100)	Mostly END, but all had neck dissections	Diagnosed as suspicious for metastasis if LNs appeared: a) > 1cm or b) Spherical in shape or b) ≥3 LNs in the first drainage area c) Rim enhancement, CN or ECS d) FDG Uptake criteria	NR	At least surgical resection, 21(100)	1. Patients with cN0 OSCC confirmed histopathologically 2. Underwent PET imaging evaluation with 18F FDG Contrast	"High spatial resolution, ease of multiplanar scanning, power Doppler, and the ability to perform guided fine-needle aspiration for cytology give ultrasound (US) an advantage over other imaging techniques."
**34**	**Righi et al. 1997**	**US**	25	USA	Retrospective	NR	Between 1993 and 1995	Mostly Upper aerodigestive tract	NR	1. MRND, 21(63.64) 2. SOND, 12(36.36)	1. Preoperative US imaging of the neck(s) at risk with a 10-MHz linear array probe was used to perform the US exams 2. All ultrasounds were performed prior to the biopsy of the primary tumor 3. Diagnosed as suspicious for metastasis if LNs appeared: a) Short axis diameter of>7 mm b) A round node is more likely to be malignant c) The more hypoechogenic d) CN or irregular margins	NR	Surgical resection, 33(100)	1. Between 1993 and 1995 2. Histopathologically proven biopsy patients of HNSCC with cN0 3. All patients underwent Unilateral or bilateral neck dissections 4. All patients underwent US or CT Imaging evaluation with Contrast	"Overall, US-FNA was comparable to CT is screening the N0 neck in our study. The choice of which modality to employ for imaging the clinically negative neck depends on a number of factors, including the location and clinical extent of the primary tumor as well as the experience and preference of the head and neck surgeon and radiologist."
		**CT**									1. Preoperative spiral CT (on an Elscint CT) with contrast 2. Scanning began from the skull base to the thoracic inlet in 5-mm increments 3. Diagnosed as suspicious for metastasis if LNs appeared: a) Short axis diameter of>8 mm b) A round node is more likely to be malignant c) The more hypoechogenic d) CN or irregular margins				
**35**	**Rodrigues et al. 2009**	**PET/CT**	44	USA	Retrospective	65 ± 17.75	Between January 2005 and July 2007	1. OP, 29(65.91) 2. HP, 1(2.27) 3. Laryngeal, 4(9.91) 4. Skin, 4(9.91) 5. Unknown, 6(13.64)	NR	Unilateral or bilateral, 44(100)	1. Performed with a commercial combined 16-slice PET/CT scanner (Biograph 16; Siemens) with 18FDG Contrast 2. Fasted for 6 h, their serum BGL was measured and found to be less than 200 mg/dL 3. Scanning was from the mid-forehead to the mid-thighs 4. Diagnosed by FDG Criteria upon a 5-point scale: (1, definitely benign; 2, probably benign; 3, equivocal; 4, probably malignant ;and 5, definitely malignant)	15	Surgical resection, 44(100)	1. Between January 2005 and July 2007 2. Consecutive patients referred for PET/CT for HN cancer Confirmed Histopathologically with N0 3. All patients were potential candidates for curative surgery 4. All patients underwent PET/CT or CT Imaging evaluation with Contrast 5. With an average age of 65 years	"The primary advantage of the dedicated HN PET/CT protocol over the WB protocol or CECT in the staging of head and neck cancer is in the detection of small lymph node metastases."
		**CT**									Diagnosed as suspicious for metastasis if LNs appeared: a) > 1cm or b) Spherical in shape or b) ≥3 LNs in the first drainage area c) Rim enhancement, CN or ECS	NR			

**36**	**Roh et al. 2014**	**PET/CT**	91	South Korea	Prospective	59 ± 9.33	From October 2010 to December 2012	1. OC, 69(75.82)<br>2. HP, 9(9.89)<br>3. Larynx, 8(8.79)<br>4. OP, 5(5.49)	Pathologically:<br>1. T1, 34(37.36)<br>2. T2, 24(26.37)<br>3. T3, 11(12.01)<br>4. T4, 22(24.18)<br>a. N0, 53(58.24)<br>b, N1, 18(19.78)<br>c. N2b, 15(1.48)<br>d. N2c, 5(5.49)	a. >1LN, MRND<br>b, Others, SND	1. Performed with a Biograph Sensation 16 or TruePoint 40 system with IV Contrast FDG<br>2. Equipped with 16- or 40-detector CT<br>2. Fasted for 6 h, their serum BGL was measured and found to be less than 150 mg/dL<br>4. Scanning was from the skull base to the upper chest<br>5. Diagnosed as suspicious for metastasis if LNs appeared: a) > 1cm or b) Spherical in shape or b) ≥3 LNs in the first drainage area<br>c) Rim enhancement, CN or ECS<br>d) During FDG Uptake criteria	2.5	1. Surgery alone, 48(52.75)<br>2. Surgery and RT, 13(14.29)<br>3. Surgery and CRT, 30(32.97)	1. From October 2010 to December 2012<br>2. Patients who had previously untreated HNSCC and no palpable lymph nodes in the neck<br>3. Each patient underwent a specific Neck dissection for his stage of disease<br>4. All patients underwent PET/CT Imaging evaluation with Contrast<br>5. At least 18 years of age	"18F-FDG PET/CT is superior to CT/MR imaging in depicting occult cervical metastatic nodes in patients with negative neck palpation findings. The improved detection and nodal staging may promote appropriate therapeutic planning in these patients."
**37**	**Salman et al. 2017**	**CT**	200	Pakistan	Prospective	44.89 ± 9.82	From October 2015 to April 2016	OC, OP, HP and Larynx, 200(100)	NR	NR	1. Performed on GE 16-slice CT scanner with contrast 2. Axial planes in coronal reconstructions<br>3. Scanning protocol was 3mm section thickness, 3mm collimation, and 3mm reconstruction interval<br>4. Diagnosed as suspicious for metastasis if LNs appeared: a) > 1cm or b) Spherical in shape or b) ≥3 LNs in the first drainage area<br>c) Rim enhancement, CN or ECS	1.33	NR	1. From October 2015 to April 2016<br>2. Histopathologically proven biopsy patients of HNSCC with cN0<br>3. Each patient underwent a specific Neck dissection for his stage of disease<br>4. All patients underwent MDCT Imaging evaluation with Contrast<br>5. Age ranges from 18 to 75 years	"MDCT is a very effective modality in early diagnosis of cancer involvement of SCC and its invasion of cervical LN metastasis promising early detection and treatment."
**38**	**Schoder et al. 2006**	**PET/CT**	31	USA	Prospective	60 ± 12	From September 2002 until November 2004	1. Tongue, 25(80.65)<br>2. Gums, 2(6.45)<br>3. FOM, 4(12.9)	Pathologically:<br>1. T1, 13(41.94)<br>2. T2, 14(45.16)<br>3. T3, 3(9.68)<br>4. T4, 1(3.23)	1. Unilateral, 26(83.87)<br>2. Bilateral, 5(16.13)<br>a. SOND , 11(35.48)<br>b. Extended SOHND, 14(45.16)<br>c. MRND, 10(32.26)<br>d. LND, 1(3.23)	1. All Patients were performed on an integrated PET/CT scanner with contrast<br>2. From the mid skull to the thoracic inlet<br>3. 5-mm scan width, 4.25mm interval in high-sensitivity mode with 15 mm per rotation<br>4. Diagnosed as suspicious for metastasis if LNs appeared: a) > 1cm or b) Spherical in shape or b) ≥3 LNs in the first drainage area<br>c) Rim enhancement, CN or ECS<br>d) During FDG Uptake criteria	Five	Mainly surgical resection	1. From September 2002 until November 2004<br>2. Patient population with newly diagnosed OSCC confirmed histopathologically with cN0<br>3. Scheduled for surgery or END<br>4. All patients underwent PET/CT imaging evaluation with Contrast<br>5. With an average age of 60 years	"18F-FDG PET/CT can identify lymph node metastases in a segment of patients with oral cancer and N0 neck. A negative test can exclude metastatic deposits with high specificity. Despite reasonably high overall accuracy; however, the clinical application of PET/CT in the N0 neck may be limited by the combination of limited sensitivity for small metastatic deposits and a relatively high number of false-positive findings. Therefore, the surgical management of the N0 neck should not be based on PET/CT findings alone."
**39**	**Schroeder et al. 2008**	**CT**	17	Germany	Prospective	56 ± 7.67	From February 2002 to June 2004	1. Tongue, 7(41.18)<br>2. Tonsils, 4(23.53)<br>2. BM and Uvula, 2(11.76)<br>3. Mouth floor, 4(23.53)	Pathologically:<br>1. T1, 6(35.29)<br>2. T2, 11(64.71)<br>a. N0, 11(64.71)<br>b. N1, 5(29.41)<br>C. N2a, 1(5.88)	END:<br>1. Ipsilateral, 7(41.8)<br>2. Bilateral, 10(58.82)	1. Axial CT scans of the primary lesion and the cervical region were performed with standard spiral CT scanners with contrast<br>2. The slice thickness was 4 mm, the tube voltage was 140 kV, and the current was 11 mA<br>3. Diagnosed as suspicious for metastasis if LNs appeared: a) > 1cm or b) Spherical in shape or b) ≥3 LNs in the first drainage area c) Rim enhancement, CN or ECS	NR	1. Surgery alone, 11(64.71)<br>2. Surgery and RT, 6(35.29)	1. From February 2002 to June 2004<br>2. Patients with oSCC staged cT1-T4 cN0-N3 cM0<br>3. All patients then underwent eND with resection of levels Ia, Ib, IIa, IIb, and III<br>4. All patients underwent Computed tomography, MRI, and 18FDG-PET<br>5. With an average age of 56 years	"The detectability threshold of occult metastases appears to be below the spatial and contrast resolution of CT, MRI, and 18FDG-PET. The decision for eND in patients with cT1-T2 cN0 cM0 OSCC cannot be based upon cross-sectional imaging at the resolutions currently available."

		**PET**									1. The 18FDG-PET images were obtained with a Siemens ECAT ExACT HR scanner<br>2. With an in-plane spatial resolution of<br>5 mm full width at half maximum at the center of the field of view<br>3. Fasted for at least 6 hours before the imaging with serum BGL≤ 150mg/dl<br>4. Diagnosed as suspicious for metastasis Upon FDG Uptake criteria				
		**MRI**									1. Performed on a 1.0T or a 1.5T whole-body scanner with contrast<br>2. Diagnosed as suspicious for metastasis if LNs appeared:<br>a) > 1cm or b) Spherical in shape or b) ≥3 LNs in the first drainage area<br>c) Rim enhancement, CN or ECS	60 post-injection			
**40**	**Sohn et al. 2015**	**PET/CT**	49	South Korea	Retrospective	59.1 ± 13.5	From July 2007 to June 2014	1. Tongue, 29(59.2)<br>2. Tonsils, 16(32.7)<br>3. PPW, 3(6.1)<br>4. Soft palate, 1(2)	Pathologically:<br>1. T1, 16 (23.65)<br>2. T2, 23(46.94)<br>3. T3, 6(12.24)<br>4. T4, 4(8.16)	1. Unilateral, 30(61.22)<br>2. Bilateral, 19(38.78)<br>(Elective or therapeutic) based on the absence or presence of metastatic lymph nodes	1. Acquired with a PET-CT unit with FDG Contrst<br>2. Fasted for at least 6 hours before the imaging with serum BGL< 140mg/dl<br>3. From the skull base to the carina<br>4. Diagnosed as suspicious for metastasis Upon FDG Uptake criteria	60 post-injection	Surgical resection, 49(100)	1. From February 2007 to June 2014<br>2. Histopathologically proven biopsy of OSCC patients with cN0<br>3. All patients underwent primary tumor resection and neck dissection<br>4. All patients underwent PET/CT imaging evaluation<br>5. With an age range of (31–85) years	"Addition of PET-CT to CT/MRI did not provide better diagnostic accuracy for detecting nodal metastasis in preoperative evaluation of oropharyngeal SCC patients with palpably negative neck, suggesting that current imaging studies might not replace elective neck dissection."
**41**	**Stevens et al. 1985**	**CT**	40	USA	Prospective	25-82	ND	ND	Pathologically:<br>≥T2, 40(100)	RND or MRND, 40(100)	1. Examined with a CT scanner (Siemens Somatom II) using a magnification with contrast<br>2. Axial scans with 4-mm section thickness and 10-s scan time<br>3. Contiguous 4-mm sections were obtained<br>4. Diagnosed as suspicious for metastasis if LNs appeared: a) > 1cm or b) Spherical in shape or b) ≥3 LNs in the first drainage area<br>c) Rim enhancement, CN or ECS	0.167	Surgical resection and/or RT, 40(100)	1. Histopathologically proven biopsy of HNSCC patients<br>2. All patients underwent RND and MRND<br>3. Underwent a prospective evaluation of the clinical radiology (CT)<br>4. With an age range of 25-82 years	"Because CT is more accurate than the clinical examination, it should be included in the staging of not only the primary tumor but also nodal disease of the neck. It can have an important role in the management of head and neck cancer."
**42**	**Stoeckli et al. 2002**	**PET**	12	Switzerland	Prospective	59.5 ± 10.5	ND	Oral, 12(100)	Pathologically:<br>1. T1, 10 (83.33)<br>2. T2, 2(16.67)	Biopsy or END, 12(100)	1. Performed with GE Advance PET scanner with FDG contrast<br>2. 2D sections in an axial field of view of 14.6 cm<br>3. Fasted for at least 4 hours<br>4. Diagnosed as suspicious for metastasis Upon FDG Uptake criteria	45 post-injection	At least surgical resection, 12(100)	1. Histopathologically proven biopsy of OSCC patients with cN0<br>2. All patients underwent RND and MRND<br>3. Underwent a prospective evaluation of the clinical radiology (CT)<br>4. With an age range of 25-82 years	"PET with FDG turned out to have a poor sensitivity and specificity in revealing occult metastasis and has no role for the evaluation of otherwise clinically N0 necks. The failure to detect micrometastasis by PET is due to the technical limitations of resolution. SLN biopsy, with END in cases of positive SLN, provides highly accurate staging of N0 necks in oral and oropharyngeal carcinoma. Patients with negative SLN could be spared the risks and the morbidity of END."

**43**	**Takes et al. 1998**	**US**	50	Netherlands	Retrospective	NR	Between 1993 and 1995	1. Larynx, 25(50)<br>2. HP, 2(4)<br>3. OP, 6(12)<br>4. OC, 17(34)	Pathologically:<br>1. T1, 2(4)<br>2. T2, 9(18)<br>3. T3, 14(28)<br>4. T4, 21(42)<br>5. Recurrence, 3(6)<br>6. Unknown, 1(2)	ND	1. Preoperative US imaging of the neck(s) at risk with a 10-MHz linear array probe was used to perform the US exams<br>2. All ultrasounds were performed prior to the biopsy of the primary tumor<br>3. Diagnosed as suspicious for metastasis if LNs appeared: a) Short axis diameter of>7 mm<br>b) A round node is more likely to be malignant<br>c) The more hypoechogenic<br>d) CN or irregular margins	NR	At least surgical resection, 50(100)	1. Between 1993 and 1995<br>2. Histopathologically proven biopsy patients of HNSCC with cN0<br>3. All patients underwent Unilateral or bilateral neck dissections<br>4. All patients underwent US or CT Imaging evaluation with Contrast	"Approximately one-half of the clinically occult nodal metastases in our patient group were identified by both CT and UGFNAB. Overall, UGFNAB and CT demonstrated comparable accuracy. The sensitivity of CT was slightly better than UGFNAB, but the latter remained characterized by a superior specificity. The results of CT and UGFNAB did not appear to be supplementary. The choice of imaging modality for staging of the clinically negative neck depends on tumor site, T-stage, and experience and preference of the head and neck oncologist. If CT is required for the staging of the primary tumor, additional staging of the neck by UGFNAB does not provide significant additional value."
		**CT**									1. Preoperative spiral CT (on an Elscint CT) with contrast<br>2. Scanning began from the skull base to the thoracic inlet in 5-mm increments<br>3. Diagnosed as suspicious for metastasis if LNs appeared: a) Short axis diameter of>8 mm<br>b) A round node is more likely to be malignant<br>c) The more hypoechogenic<br>d) CN or irregular margins	NR			
**44**	**Thomsen et al. 2005**	**US**	40	Denmark	Prospective	32–90	ND	Oral, 40(100)	Pathologically:<br>1. T1, 24(60)<br>2. T2, 16(40)	Variable Neck dissections, 40(100)	1. Used a Siemens Sonoline Elegra ultrasonography with a 7.5 MHz linear-array transducer<br>2. Diagnosed as suspicious for metastasis if LNs appeared: a) Short axis diameter of>7 mm<br>b) A round node is more likely to be malignant<br>c) The more hypoechogenic<br>d) CN or irregular margins	NR	Surgical resection or biopsy	1. Consecutive patients with OSCC confirmed histo-pathologically with cN0<br>2. All patients had surgical resection and/or biopsy<br>3. Underwent US or MRI imaging evaluation<br>4. With an age range of 32-90 years	"Sentinel lymph node biopsy improved staging of patients with small N0 oral cancers. Combined sentinel lymph node biopsy and Doppler ultrasonography may further improve staging. MRI and simple palpation results were poor."
		**MRI**									1. Performed on a 1.5T system with Gadolinium contrast<br>2. Slice thickness was 6 mm,<br>3. Scanned from the base of the cranium to the sternoclavicular joint<br>4. Diagnosed as suspicious for metastasis if LNs appeared: a) > 1cm or b) Spherical in shape or b) ≥3 LNs in the first drainage area<br>c) Rim enhancement, CN or ECS				
**45**	**To et al. 2003**	**US**	30	Hong Kong	Retrospective	Mean (53.4)	Between January 1985 and December 2000	Tongue, 30(100)	Pathologically (T1 and T2)	END, 30(100)	1. Performed using a real-time high-resolution scanner with a linear 7.5 – 10 MHz frequency probe<br>2. Scanning was from above the clavicle to the submental and retroparotideal regions<br>3. Diagnosed as suspicious for metastasis if LNs appeared: a) Short axis diameter of>7 mm<br>b) A round node is more likely to be malignant<br>c) The more hypoechogenic<br>d) CN or irregular margins	NR	At east surgical resection, 30(100)	1. Between January 1985 and December 2000<br>2. Consecutive patients with OSCC confirmed histo-pathologically with cN0<br>3. All patients had surgical resection<br>4. Underwent US imaging evaluation<br>5. With an average age of 53.4 years	"It is concluded that ultrasound alone is inadequate for making decisions regarding neck management of patients with T1 and T2 N0 carcinoma of the tongue and cannot replace a policy of elective neck dissection."

46	**Tuli et al. 2008**	**CT**	20	India	Prospective	54.75 ± 14.57	ND	Tongue, 20(100)	Clinically (T1 and T2),	Functional Neck dissection, 20(100)	1. Performed using single-slice spiral CT and Non-ionic contrast While MRI was performed using 1.5 T Magnetron<br>2. Scanning was from above the clavicle to the submental and retroparotideal regions<br>3. Diagnosed as suspicious for metastasis if LNs appeared: a) > 1cm or b) Spherical in shape or b) ≥3 LNs in the first drainage area<br>c) Rim enhancement, CN or ECS	NR	Wide local excision of the primary tumor followed by functional neck dissection	1. Histopathologically proven carcinoma of the tongue and clinically neck-negative (T1–T2N0)<br>2. Scheduled for biopsy and/or surgical resection<br>3. Underwent CT or MRI imaging evaluation<br>4. With an average age of 54.75 years	"In this preliminary prospective study, we observed that 40% (8/20) of the clinically neck-negative carcinoma tongue patients harbored metastatic LNs. 99mTc-MIBI-SPECT is a more effective imaging modality in the staging of clinically neck-negative LN metastases in carcinoma tongue as compared with CT or MR."
		**MRI**													
47	**Van den brekel et al. 1993**	**US**	132	Netherlands	Prospective	NR	From December 1988 until July 1990	1. OC, 47(35.61)<br>2. OP, 39(29.55)<br>3. HP, 13(9.85)<br>4. Larynx, 29(21.97)<br>5. Other, 4(3.3)	Pathologically:<br>1. T1, 11(8.33)<br>2. T2, 36(27.27)<br>3. T3, 57(34.18)<br>4. T4, 28(21.21)	1. RND , 142(78.89)<br>2. SND, 38(21.11)	Diagnosed as suspicious for metastasis if LNs appeared: a) Short axis diameter of>7 mm<br>b) A round node is more likely to be malignant<br>c) The more hypoechogenic<br>d) CN or irregular margins	Surgical resection, 180(100)	At least surgical resection, 132(100)	1. From December 1988 until July 1990<br>2. Previously untreated patients with SCC of the upper aerodigestive tract<br>3. Underwent neck dissection as part of their treatment<br>4. All patients were examined with preoperative MRI, CT and US	"The accuracy of US-guided aspiration cytology was significantly better than of any other technique used in this study. Modern imaging techniques are essential for the appropriate assessment of neck node metastases. In view of advances in the accuracy of contemporary imaging, the need for elective treatment of the neck requires reappraisal."
		**MRI**									Diagnosed as suspicious for metastasis if LNs appeared: a) > 1cm or b) Spherical in shape or b) ≥3 LNs in the first drainage area<br>c) Rim enhancement, CN or ECS				
		**CT**													
48	**Vartak et al. 2023**	**PET/CT**	51	India	Prospective	54.69 ± 12.5	From May 2015 to Apr 2017	1. Tongue, 27(52.94)<br>2. BM, 15(29.41)<br>3. Lips, 4(7.84)<br>4. Mouth floor, 2(3.92)<br>5. LA, 1(1.96)<br>6. UA, 1(1.96)<br>7. RA, 1(1.96)	Pathologically:<br>1. T1, 31(60.78)<br>2. T2, 20(39.22)	By SOND:<br>1. Unilateral, 44(86.27)<br>2. Bilateral, 7(13.73)	1. Performed with a dedicated PET/CT scanner with a LSO (Lutetium oxyorthosilicate $Li_2SiO_5:Ce$) detector with contrast<br>2. Diagnosed as suspicious for metastasis Upon FDG Uptake criteria	Two	Surgical resection, 51(100)	1. From May 2015 to Apr 2017<br>2. Patients with histologically proven carcinoma oral cavity who were staged as cT1–T2, cN0<br>3. Underwent Elective neck dissection as part of their treatment<br>4. All patients were examined with preoperative PET/CT<br>4. With an average age of 54.69 years	"Based on diagnostic accuracy and high negative predictive value, incorporating 18F-FDG PET/CT in preoperative staging paradigm of cT1/T2 carcinoma oral cavity will guide in the selection of patients in which cN0 neck can be safely observed."
49	**Wensing et al. 2006**	**PET**	30	Netherlands	Prospective	60 ± 13	Between June 2001 and December 2003	Oral, 30(100)	Pathologically:<br>1. T1, 9(30)<br>2. T2, 13(43.33)<br>3. T3, 4(13.33)<br>4. T4, 2(6.67)	SOND, 30(100)	1. A dedicated PET scanner with FDG contrast<br>2. Fasted for 6 hours before the imaging<br>3. Diagnosed as suspicious for metastasis Upon FDG Uptake criteria	10	At least surgical resection, 30(100)	1. Between June 2001 and December 2003<br>2. Patients with cN0 histologically proven carcinoma oral cavity<br>3. Underwent Elective neck dissection as part of their treatment<br>4. All patients were examined with preoperative PET imaging evaluation<br>5. With an average age of 60 years	"In patients with cN0 SCC of the oral cavity, FDG-PET does not contribute to the preoperative workup. FDG-PET does not replace SOHND as a staging procedure. Key Words: Oral cavity, squamous cell carcinoma, cervical lymph node metastasis, FDG-PET, supraomohyoid neck dissection."
50	**Wensing et al. 2011**	**US**	9	Netherlands	Prospective	≥18, 9(100%)	Between June 2005 and January 2007	OC, 9(100)	T1 and T2, 9(100)	SND, 9(100)	1. Performed using a linear 7.5 MHz probe<br>2. Diagnosed as suspicious for metastasis if LNs appeared: a) Short axis diameter of>5 mm<br>b) A round node is more likely to be malignant<br>c) The more hypoechogenic<br>d) CN or irregular margins	NR	Surgical resection, 9(100)	1. Between June 2005 and January 2007<br>2. Patients with cN0 histologically proven carcinoma oral cavity cN0<br>3. Underwent Selective neck dissection as part of their treatment<br>4. All patients were examined with preoperative US, MRI and/or CT imaging evaluation	"This pilot study shows that MRL has a high NPV based on a node-to-node analysis. However, its PPV was only 10%, and therefore, its use as a single imaging technique in the preoperative staging of the cN0 neck in SCCOC seems to be limited. Further studies are needed to confirm these data."

		**MRI**									1. Examinations were carried out at a field strength of 1.5 and 3.0 Tesla with contrast<br>2. The scanned area extended from the clivus to the aortic arch<br>3. Diagnosed as suspicious for metastasis if LNs appeared: a) > 1cm or b) Spherical in shape or b) ≥3 LNs in the first drainage area<br>c) Rim enhancement, CN or ECS			5. Aged 18 years or older of either sex<br>6. Gave an informed consent	
		**CT**									1. An MDCT scan (Siemens, Erlangen, Germany) was performed<br>2. From the anterior skull base to the aortic arch in 2 mm reconstructions<br>3. Diagnosed as suspicious for metastasis if LNs appeared: a) > 1cm or b) Spherical in shape or b) ≥3 LNs in the first drainage area<br>c) Rim enhancement, CN or ECS	0.67			
**51**	**Wilson et al. 1994**	**MRI**	42	UK	Prospective	NR	From December 1991 to March 1993	HN, 42(100)	NR	SOND, 42(100)	1. Acquired an IGE MR Max Plus 0.5T Scanner<br>2. The slice thickness was 5-7 mm with an interslice gap of 1-2 mm<br>3. Diagnosed as suspicious for metastasis if LNs appeared: a) > 1cm or b) Spherical in shape or b) ≥3 LNs in the first drainage area<br>c) Rim enhancement, CN or ECS	Six	Surgical resection, 42(100)	1. From December 1991 to March 1993<br>2. Histo-pathologically proven biopsy patients of HNSCC<br>3. Scheduled for surgery with SOND<br>4. All patients were examined with preoperative MRI imaging evaluation	"Routine use of MRI scans will allow an unnecessary neck dissection to be avoided in a patient with a clinically negative neck who also has a negative MRI scan."
**52**	**Xu et al. 2020**	**MRI**	151	China	Retrospective	57.1 ± 8	From January 2010 to December 2016	Tongue, 151(100)	Clinically: (T1), 151(100)	END, 151(100)	1. MRI scanning (SIEMENS Prasma, 3.0T) without contrast<br>2. The images were reconstructed with the thickness of a 1.0-mm slice<br>3. Diagnosed as suspicious for metastasis if LNs appeared: a) > 1cm or b) Spherical in shape or b) ≥3 LNs in the first drainage area<br>c) Rim enhancement, CN or ECS	NR	Surgical resection, 151(100)	1. From January 2010 to December 2016<br>2. Patients with surgically treated tongue SCC with cN0 confirmed histo-pathologically<br>3. Scheduled for surgery with END<br>4. All patients were examined with preoperative MRI imaging evaluation<br>5. Adult (≥18 years old)	"In summary, there is a significant relationship between MRI-determined DOI and occult neck lymph node metastasis in cT1N0 tongue SCC and elective neck dissection and adjuvant therapy are suggested if MRI-determined DOI is greater than 7.5 mm; MRI-determined DOI ≥ 7.5 mm indicates additional risk for disease recurrence and cancer-related death."
**53**	**Yamaga et al. 2018**	**PET/CT**	205	Japan	Retrospective	59.7 ± 14.9	Between June 2010 and December 2014	1. Tongue, 135(65.9)<br>2. UA, 3(11.2)<br>3. LA, 32(15.6)<br>4. BM, 9(4.4)<br>5. Mouth floor, 5(2.4)<br>6. Hard palate, 1(0.5)	Clinically:<br>1. T1, 88(42.9)<br>2. T2, 117(57.1)	Neck dissection, 61(29.8)	1. A dedicated PET-scanner with FDG contrast<br>2. Fast for at least 4 h before receiving an injection of FDG<br>3. Scanning was from the skull base to subclavicular area<br>4. Diagnosed as suspicious for metastasis if LNs appeared: a) > 1cm or b) Spherical in shape or b) ≥3 LNs in the first drainage area<br>c) Rim enhancement, CN or ECS<br>d) Upon FDG Uptake criteria	Four	1. Surgical resetion, 188(91.7)<br>2. Brachytherapy, 16(7.8)<br>3. PBT and IA Chemo.T, 1(0.5)	1. Between June 2010 and December 2014<br>2. Consecutive patients with early OSCC (cT1-2N0M0) confirmed histo-pathologically<br>3. Underwent FDG-PET/CT prior to treatment<br>4. With an average age of 59.7 years	"Although its utility for detecting cervical nodal metastases and synchronous cancers is limited, FDG-PET/CT is a potentially prognostic indicator in early OSCC."

54	**Yamane et al. 2007**	**US**	109	Japan	Prospective	57 ± 11.5	Between 1998 and 2002	Tongue, 109(100)	Clinically:<br>(T1 and T2), 109(100)	END(N0) or MRND , 109(100)	1. The ultrasound images were acquired using an Aloka SSD-630 ultrasound system<br>2. A 10-MHz mechanical sector transducer was used<br>3. From the surface to the maximal thickness of the tumor, the accuracy was 0.1 mm<br>4. Diagnosed as suspicious for metastasis if LNs appeared: a) Short axis diameter of>7 mm<br>b) A round node is more likely to be malignant<br>c) The more hypoechogenic<br>d) CN or irregular margins	Two	Surgical resection, 109(100)	1. Between 1998 and 2002<br>2. Consecutive patients with OSCC confirmed histo-pathologically<br>3. All patients are studied using US imaging evaluation<br>4. With an average age of 57 years<br>5. Gave an informed consent	"Intraoral ultrasonography in conjunction with the proposed CAD system allows tissue characterization and prediction of subclinical cervical lymph node metastasis."
55	**Yucel et al. 1997**	**MRI**	18	Turkey	Retrospective	60 ± 10.5	NR	1. Larynx, 13(72.77)<br>2. Mouth floor, 1(5.56)<br>3. Tongue, 3(16.67)<br>4. Pyriform sinus, 1(5.56)	Pathologically:<br>1. T1, 1(5.56)<br>2. T2, 6(33.33)<br>3. T3, 11(61.11)	Variable Neck dissections, 18(100)	1. Performed with a 0.5 T image<br>2. The section thickness was 5mm, and the slice gap was 0.5 mm for both axial and coronal images<br>3. Covering the region from the mandible to the sternal<br>4. Diagnosed as suspicious for metastasis if LNs appeared: a) > 1cm or b) Spherical in shape or b) ≥3 LNs in the first drainage area<br>c) Rim enhancement, CN or ECS	NR	Surgical resection, 18(100)	1. Consecutive patients with cN0 HNSCC confirmed histo-pathologically<br>2. All patients are studied using MRI imaging evaluation<br>3. With an average age of 56 years	"MR may reveal metastatic lymph nodes in patients with no clinical evidence of metastasis. However, conventional MR techniques are not always sufficient for decision-making on surgery in cases of (No neck)."
56	**Zhang et al. 2018**	**PET/CT**	96	Canada	Retrospective	58.1 ± 13.8	Between 2009 and 2013	1. Tongue, 22(68.8)<br>2. FOM, 9(28.1)<br>3. BM, 3(9.4)<br>4. UA/LA, 1(3.1)<br>5. RA, 1(3.1)	Clinically (T1 and T2), 32(100)	1. Unilateral:<br>a. SND,17(47.2)<br>b. RND, 1(2.8)<br>2. Bilateral:<br>SND, 16(50)	1. Radiographic imaging in the form of PET-CT or CT Neck with FDG contrast for staging of the neck<br>2. Diagnosed as suspicious for metastasis if LNs appeared:<br>a) > 1cm or b) Spherical in shape,<br>b) ≥3 LNs in the first drainage area,<br>c) Rim enhancement, CN or ECS, or<br>d) Upon FDG Uptake criteria	NR	Surgical resection, 32100)	1. Between 2009 and 2013<br>2. Adult OCSCC patients with cN0 cT1 or cT2 tumors<br>3. Underwent preoperative PET-CT and CT Neck with Contrast<br>4. With an average age of 58.1 years	"In patients with cT1 and T2N0 OCSCC, PET-CT has high negative predictive value. These patients can be considered for treatment with single modality surgical resection and elective neck dissection."
57	**Zhao et al. 2020**	**PET/CT**	135	China	Prospective	54.5 ± 7.67	From January 2010 to December 2018	Tongue, 135(100)	Clinically (T1 and T2), 135(100)	END, 135(100)	1. Several PET/CT scanners were used to perform the PET-CT scans<br>2. Fasted for at least 6 hours before the imaging with serum BGL <200mg/dl<br>3. From the calvarial vertex through the upper thighs after urinary voiding<br>4. The images were reconstructed in the thickness of a 2.5 mm slice<br>5. Diagnosed as suspicious for metastasis if LNs appeared: a) > 1cm or b) Spherical,<br>b) ≥3 LNs in the first drainage area,<br>c) Rim enhancement, CN or ECS, or<br>d) Upon FDG Uptake criteria	NR	Surgical resection, 135(100)	1. From January 2010 to December 2018<br>2. Patients with primary early-stage (cT1-2N0) tongue SCC<br>3. Agreed to undergo a PET-CT examination preoperatively<br>4. With an average age of 54.5 years<br>5. Gave an informed consent	"PET-CT has a high specificity for predicting occult lymph node metastasis, and an SUV max >9.0 is significantly associated with worse LRC in cT1-2N0 tongue SCC."

Abbreviations: ND= Not Determined; NR= Not Reported; SD= Standard Deviation; MRI= Magnetic Resonance Imaging; DWI= Diffuse Weight Imaging; US= Ultra-Sonography; FNAC= Fine Needle Aspiration Cytology; PET= Positron Emission Tomography; FDG= Flouro-Deoxy Glucose; CT= Computed Tomography; SPECT= Single Photon Emission Tomography; HN= Head and Neck; OC= Oral cavity; HNSCC= Head and Neck Squamous Cell Carcinoma; OSCC= Oral Squamous Cell Carcinoma; OP= Oro-Pharynx= HP= Hypo-Pharynx; SG= Sub-Glottis; PNSs= Para-Nasal Sinuses; LA= Lower Alveolus; UA= Upper Alveolus; RA= Retromolar Area; FOM= Floor Of Mouth; BM= Buccal Mucosa; BGL= Blood Glucose Level; RND= Radical Neck Dissection; MRCND= Modified Radical Neck Dissection; SND= Selective Neck Dissection; SOND= Supra-Omohyoid Neck Dissection; END= Elective Neck Dissection; ECS= Extra Capsular Spread; CN= Central Necrosis
