# Supplementary material for: Comparing the Efficacy of CT, MRI, PET-CT, and US in the Detection of Cervical Lymph Node Metastases in Head and Neck Squamous Cell Carcinoma with Clinically Negative Neck Lymph Node: A Systematic Review and Meta-Analysis
Source: J Clin Med. 2024 Dec 14;13(24):7622. doi: 10.3390/jcm13247622 (PMC11728035; doi:10.3390/jcm13247622)
Supplement: Supplementary file 1 [file jcm-13-07622-s001.zip › Supplementary Table S2.pdf]

Study ID	Modality	Risk of bias: Patient selection 1	2	3	Index test 4	Reference standard 5	6	7	Flow and timing 8	9	ROB Summary Result	Applicability: Patient selection 1	Index test 2	Reference standard 3	Applicability Summary Result
Akoglu et.al 2005	US	Y	Y	Y	U	Y	U	Y	Y	Y	B	Y	Y	Y	H
	CT	Y	Y	Y	U	Y	U	Y	Y	Y	B	Y	Y	Y	H
	MRI	Y	Y	Y	U	Y	U	Y	Y	Y	B	Y	Y	Y	H
Bae et al 2019	PET/CT	U	Y	Y	Y	Y	Y	Y	Y	Y	C	Y	Y	Y	H
Barchetti et.al 2014	MRI	Y	Y	Y	Y	Y	N	Y	Y	Y	B	Y	Y	Y	H
Bhargava et.al 2015	CT	U	Y	Y	Y	Y	U	U	Y	Y	B	Y	Y	Y	H
Braams et.al 1995	PET	U	Y	Y	Y	Y	U	U	Y	Y	B	Y	Y	Y	H
	MRI	N	Y	Y	Y	Y	U	Y	Y	Y	C	Y	Y	Y	H
Brouwer et.al 2003	CT	Y	Y	Y	Y	Y	Y	Y	Y	N	B	Y	Y	Y	H
	PET	Y	Y	Y	Y	Y	Y	Y	Y	Y	C	Y	Y	Y	H
	US	Y	Y	Y	Y	Y	Y	Y	Y	N	B	Y	Y	Y	H
	MRI	Y	Y	Y	Y	Y	Y	Y	Y	N	B	Y	Y	Y	H
Byers et.al 1998	US	Y	Y	Y	U	Y	U	Y	Y	Y	B	Y	Y	Y	H
	CT	Y	Y	Y	U	Y	U	Y	Y	N	A	Y	Y	Y	H
Cebeci et.al 2023	MRI	U	Y	Y	Y	Y	U	Y	Y	Y	B	Y	Y	Y	H
	PET	U	Y	Y	Y	Y	U	Y	Y	Y	B	Y	Y	Y	H
Cetin et.al 2013	PET/CT	Y	Y	Y	Y	Y	N	Y	Y	Y	B	Y	Y	Y	H
Chauhan et.al 2012	CT	U	Y	Y	U	Y	U	Y	Y	Y	B	Y	Y	Y	H
	US	U	Y	Y	U	Y	U	Y	Y	Y	B	Y	Y	Y	H
	PET/CT	U	Y	Y	U	Y	U	Y	Y	Y	B	Y	Y	Y	H
Chaukar et.al 2014	US	U	Y	Y	Y	Y	U	Y	Y	Y	B	Y	Y	Y	H
	CT	U	Y	Y	Y	Y	U	Y	Y	Y	B	Y	Y	Y	H
Ding et.al 2021	US	U	Y	N	U	Y	U	Y	Y	Y	A	Y	Y	Y	H
Dresel et.al 2003	PET/CT	U	Y	Y	U	Y	U	Y	Y	Y	B	Y	Y	Y	H
	MRI	U	Y	Y	U	Y	U	Y	Y	N	A	Y	Y	Y	H
	CT	U	Y	Y	U	Y	U	Y	Y	N	A	Y	Y	Y	H
Dudau et.al 2014	US	Y	Y	Y	Y	Y	U	Y	Y	Y	C	Y	Y	Y	H
Eida et.al 2003	CT	U	Y	U	U	Y	U	Y	Y	Y	B	Y	Y	Y	H
	US	U	Y	U	U	Y	U	Y	N	Y	A	Y	Y	Y	H
Freire et.al 2003	CT	U	Y	Y	U	Y	U	Y	Y	Y	B	Y	Y	Y	H
Hodder et.al 2000	US	U	Y	U	U	Y	U	Y	Y	Y	B	Y	Y	Y	H
Horváth et.al 2020	CT	U	Y	U	Y	Y	U	Y	Y	Y	B	Y	Y	Y	H
	US	U	Y	U	Y	Y	U	Y	Y	N	A	Y	Y	Y	H

**Iyer et.al 2010**	**PET/CT**	U	Y	Y	U	Y	U	Y	Y	Y	B	Y	Y	Y	H
**Kau et.al 1999**	**PET**	Y	Y	U	Y	Y	Y	Y	Y	Y	C	Y	Y	Y	H
	**CT**	Y	Y	U	Y	Y	Y	Y	Y	N	A	Y	Y	Y	H
	**MRI**	Y	Y	U	Y	Y	Y	Y	Y	N	A	Y	Y	Y	H
**Kawano et.al 2022**	**US**	Y	Y	U	U	Y	U	Y	Y	Y	B	Y	Y	Y	H
**Konishi et.al 2022**	**US**	Y	Y	Y	U	Y	U	Y	Y	Y	B	Y	Y	Y	H
**Krabbe et.al 2008**	**CT**	Y	Y	Y	U	Y	Y	Y	Y	Y	C	Y	Y	Y	H
	**MRI**	Y	Y	Y	U	Y	Y	Y	Y	N	A	Y	Y	Y	H
	**PET**	Y	Y	Y	U	Y	Y	Y	Y	N	A	Y	Y	Y	H
**Lee et.al 2015**	**PET/CT**	Y	Y	Y	Y	Y	Y	Y	Y	Y	C	Y	Y	Y	H
**Madsen et.al 2023**	**PET/CT**	Y	Y	Y	U	Y	Y	Y	Y	Y	C	Y	Y	Y	H
	**MRI**	Y	Y	Y	U	Y	Y	Y	Y	Y	C	Y	Y	Y	H
**Maremonti et.al 1997**	**US**	Y	Y	Y	N	Y	N	Y	Y	Y	A	Y	Y	Y	H
	**MRI**	Y	Y	Y	N	Y	N	Y	Y	Y	A	Y	Y	Y	H
	**CT**	Y	Y	Y	N	Y	N	Y	Y	Y	A	Y	Y	Y	H
**Mayordomo et.al 2016**	**US**	U	Y	Y	U	Y	U	Y	Y	Y	B	Y	Y	Y	H
**Myers et.al 1998**	**PET**	Y	Y	Y	U	Y	U	Y	Y	Y	B	Y	Y	Y	H
	**CT**	Y	Y	Y	U	Y	U	Y	Y	N	A	Y	Y	Y	H
**Nahmias et.al 2007**	**US**	Y	Y	Y	Y	Y	Y	Y	Y	Y	C	Y	Y	Y	H
**Ng 2006**	**PET**	U	Y	Y	Y	Y	Y	Y	Y	Y	C	Y	Y	Y	H
**Norling et.al 2014**	**US**	Y	Y	Y	Y	Y	Y	Y	Y	Y	C	Y	Y	Y	H
**Ozer et.al 2012**	**PET/CT**	U	Y	U	U	Y	U	Y	Y	Y	B	Y	Y	Y	H
**Richards et.al 2007**	**US**	U	Y	Y	U	Y	U	Y	Y	Y	B	Y	Y	Y	H
**Righi et.al 1997**	**US**	U	Y	Y	Y	Y	Y	Y	Y	Y	C	Y	Y	Y	H
	**CT**	U	Y	Y	Y	Y	Y	Y	Y	Y	C	Y	Y	Y	H
**Rodrigues et.al 2009**	**PET/CT**	Y	Y	Y	Y	Y	Y	Y	Y	Y	C	Y	Y	Y	H
	**CT**	Y	Y	Y	Y	Y	Y	Y	Y	Y	C	Y	Y	Y	H
**Roh et.al 2014**	**PET/CT**	Y	Y	Y	Y	Y	Y	Y	Y	Y	C	Y	Y	Y	H
**Salman et.al 2017**	**CT**	Y	Y	Y	Y	Y	Y	Y	Y	Y	C	Y	Y	Y	H
**Schoder et.al 2006**	**PET/CT**	Y	Y	Y	N	Y	N	Y	Y	Y	C	Y	Y	Y	H
**Schroeder et.al 2008**	**CT**	U	Y	Y	Y	Y	Y	Y	Y	N	A	Y	Y	Y	H
	**PET**	U	Y	Y	Y	Y	Y	Y	Y	Y	C	Y	Y	Y	H
	**MRI**	U	Y	Y	Y	Y	Y	Y	Y	Y	C	Y	Y	Y	H
**Sohn et.al 2015**	**PET/CT**	Y	Y	Y	Y	Y	Y	Y	Y	Y	C	Y	Y	Y	H
**Stevens et.al 1985**	**CT**	U	Y	U	Y	Y	U	Y	Y	Y	C	Y	Y	Y	H
**Stoeckli et.al 2002**	**PET**	Y	Y	Y	U	Y	U	Y	Y	Y	C	Y	Y	Y	H
**Takes et.al 1998**	**US**	U	Y	Y	U	Y	U	Y	Y	Y	B	Y	Y	Y	H

	**CT**	U	Y	Y	U	Y	U	Y	Y	Y	B	Y	Y	Y	H
**Thomsen et.al 2005**	**US**	Y	Y	Y	U	Y	U	Y	Y	Y	B	Y	Y	Y	H
	**MRI**	Y	Y	Y	U	Y	U	Y	Y	N	A	Y	Y	Y	H
**To et.al 2003**	**US**	U	Y	Y	U	Y	U	Y	Y	Y	B	Y	Y	Y	H
**Tuli et.al 2008**	**CT**	U	Y	Y	Y	Y	Y	Y	Y	Y	C	Y	Y	Y	H
	**MRI**	U	Y	Y	Y	Y	Y	Y	Y	Y	C	Y	Y	Y	H
**Van den brekel et.al 1993**	**US**	U	Y	Y	Y	Y	Y	Y	Y	Y	C	Y	Y	Y	H
	**MRI**	U	Y	Y	Y	Y	Y	Y	Y	N	A	Y	Y	Y	H
	**CT**	U	Y	Y	Y	Y	Y	Y	Y	N	A	Y	Y	Y	H
**Vartak et.al 2023**	**PET/CT**	U	Y	Y	Y	Y	Y	Y	Y	Y	C	Y	Y	Y	H
**Wensing et.al 2006**	**PET**	U	Y	Y	U	Y	U	Y	Y	Y	B	Y	Y	Y	H
**Wensing et.al 2011**	**US**	Y	Y	Y	Y	Y	Y	Y	Y	Y	C	Y	Y	Y	H
	**MRI**	Y	Y	Y	Y	Y	Y	Y	Y	Y	C	Y	Y	Y	H
	**CT**	Y	Y	Y	Y	Y	Y	Y	Y	Y	C	Y	Y	Y	H
**Wilson et.al 1994**	**MRI**	Y	Y	U	U	Y	U	Y	Y	Y	B	Y	Y	Y	H
**Xu et.al 2020**	**MRI**	U	Y	Y	Y	Y	Y	Y	Y	Y	C	Y	Y	Y	H
**Yamaga et.al 2018**	**PET/CT**	Y	Y	Y	U	Y	U	Y	Y	Y	B	Y	Y	Y	H
**Yamane et.al 2007**	**US**	U	Y	Y	U	Y	U	Y	Y	Y	B	Y	Y	Y	H
**Yucel et.al 1997**	**MRI**	U	Y	Y	U	Y	U	Y	Y	Y	B	Y	Y	Y	H
**Zhang et.al 2018**	**PET/CT**	U	Y	Y	U	Y	U	Y	Y	Y	B	Y	Y	Y	H
**Zhao et.al 2020**	**PET/CT**	Y	Y	Y	U	Y	U	Y	Y	Y	B	Y	Y	Y	H

Abbreviations: Y, Yes; U, Unclear; N= No; A= high risk of bias; B= Unclear risk of bias; C= Low risk of bias; H= High applicability.
