# Supplementary figures and images for: Comparing the Efficacy of CT, MRI, PET-CT, and US in the Detection of Cervical Lymph Node Metastases in Head and Neck Squamous Cell Carcinoma with Clinically Negative Neck Lymph Node: A Systematic Review and Meta-Analysis
Source: J Clin Med. 2024 Dec 14;13(24):7622. doi: 10.3390/jcm13247622 (PMC11728035; doi:10.3390/jcm13247622)

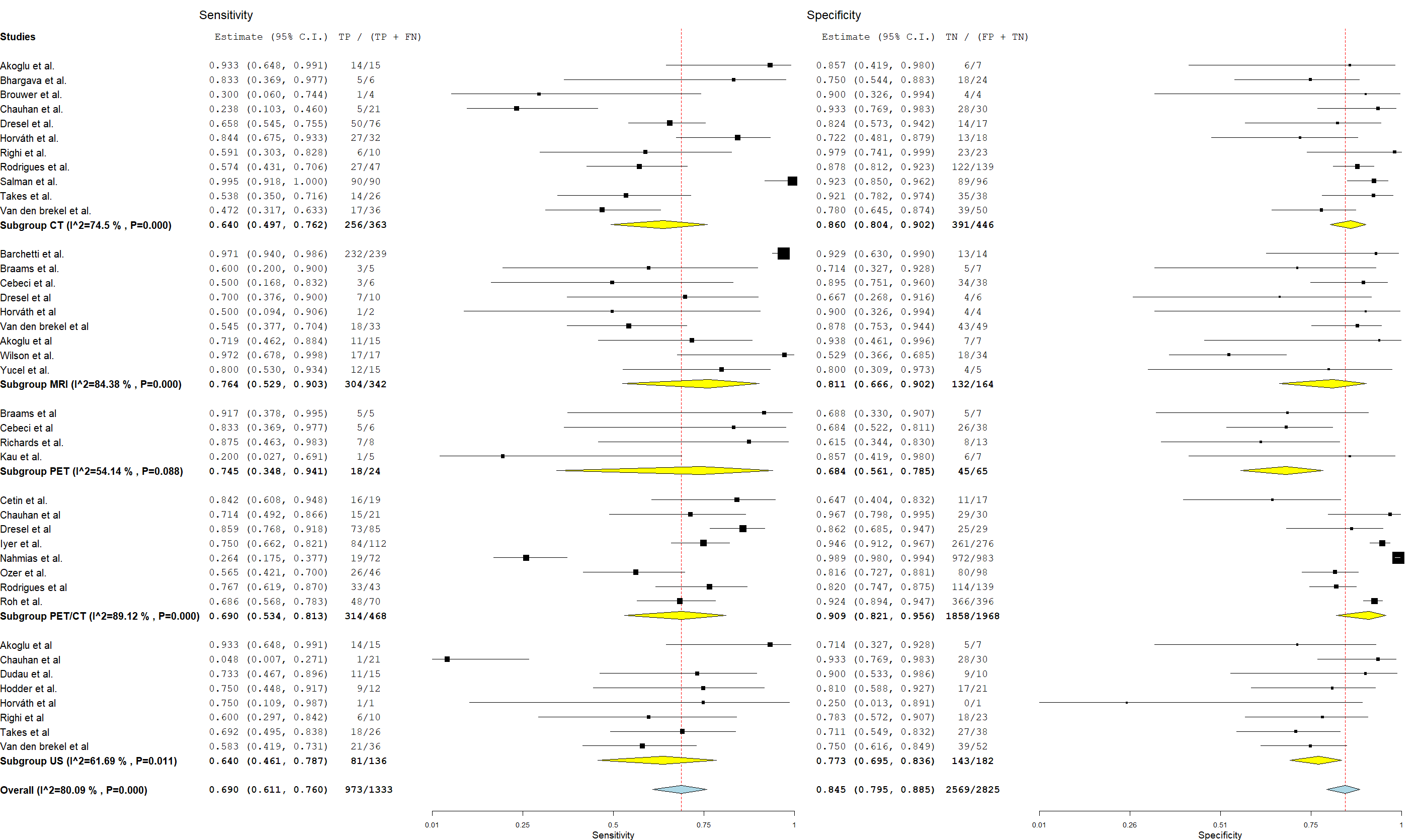

Supplement: Supplementary file 1 [file jcm-13-07622-s001.zip › Supplementary Figure S1.png]

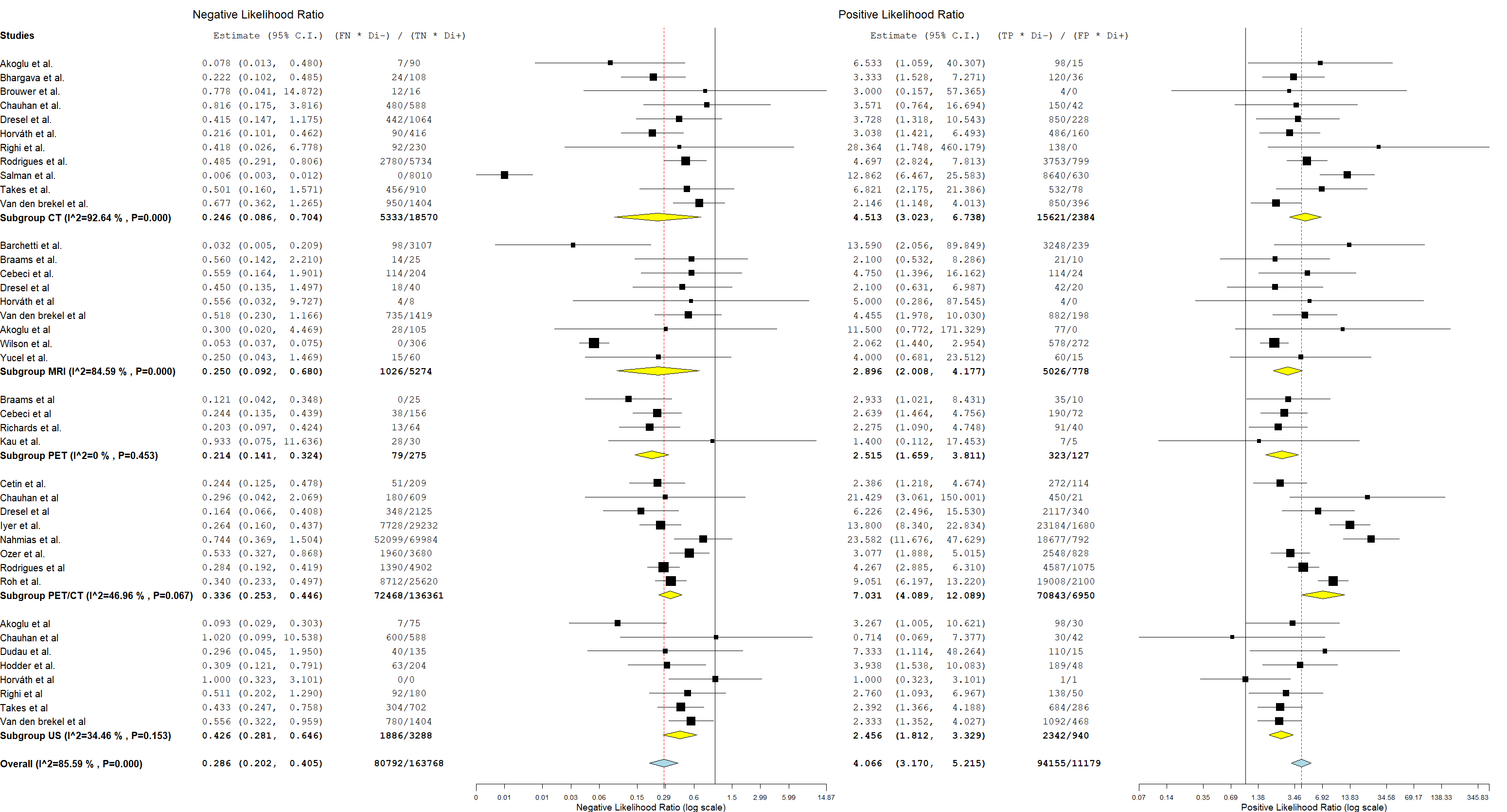

Supplement: Supplementary file 1 [file jcm-13-07622-s001.zip › Supplementary Figure S2.png]

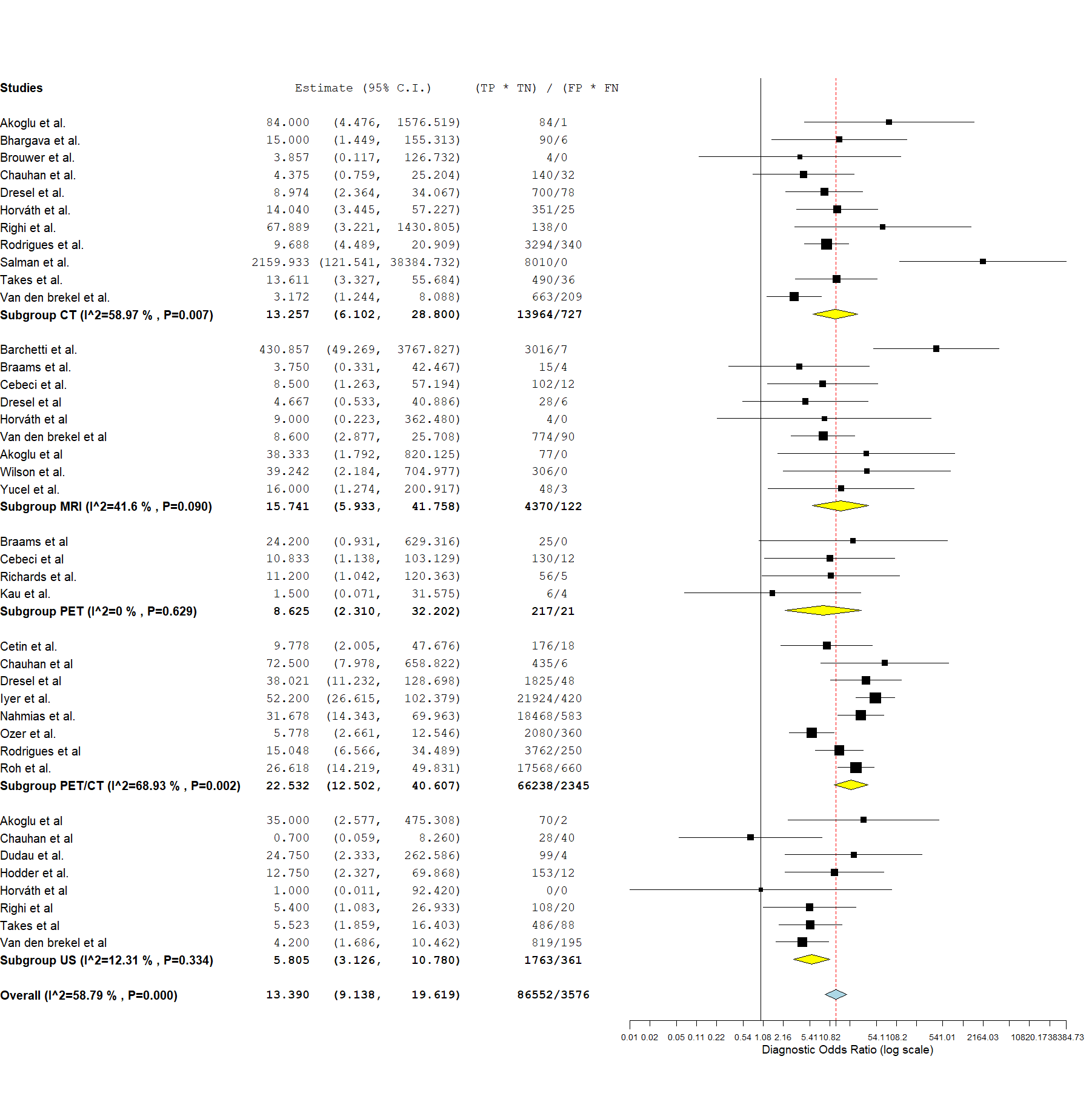

Supplement: Supplementary file 1 [file jcm-13-07622-s001.zip › Supplementary Figure S3.png]

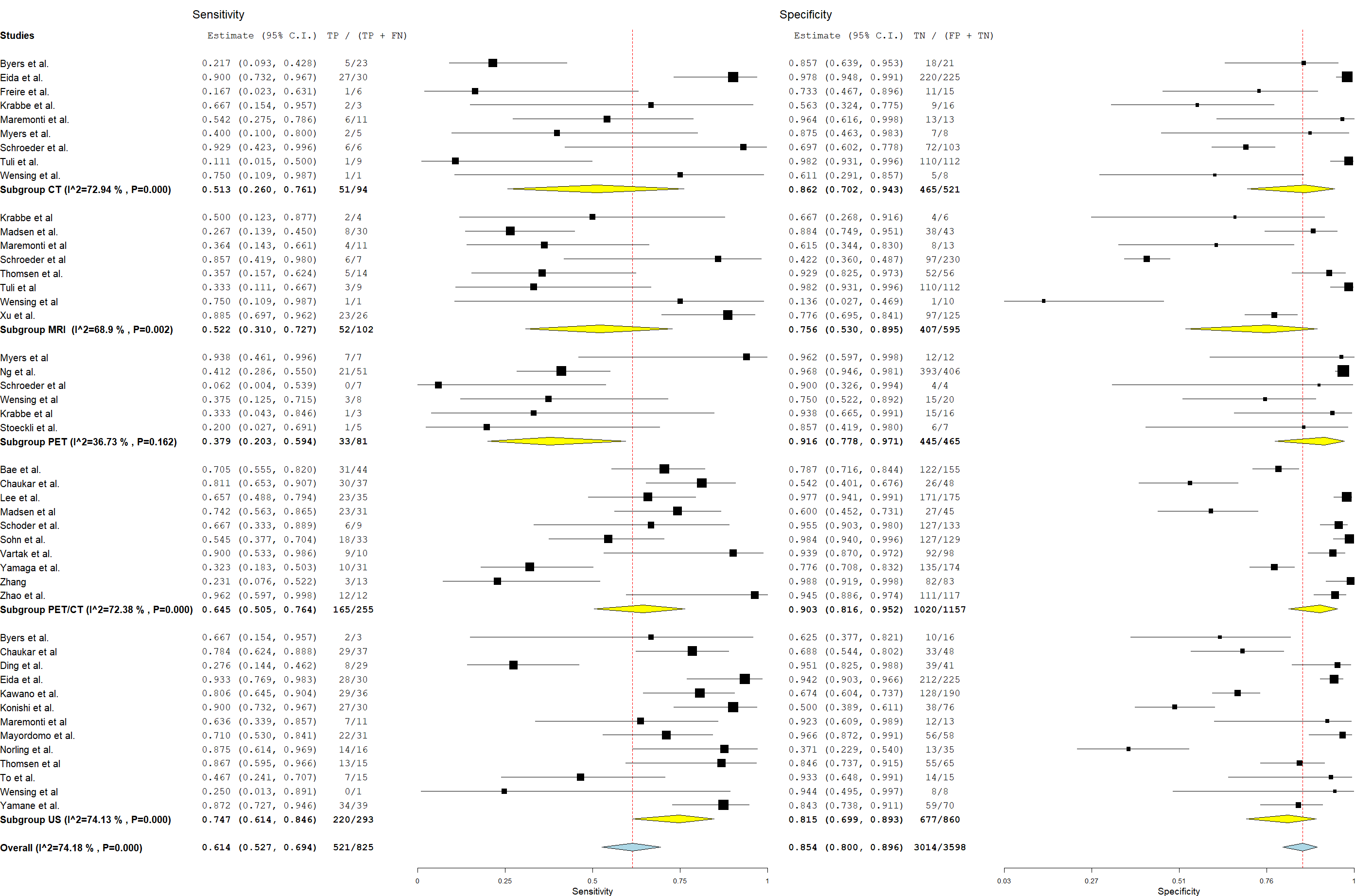

Supplement: Supplementary file 1 [file jcm-13-07622-s001.zip › Supplementary Figure S4.png]

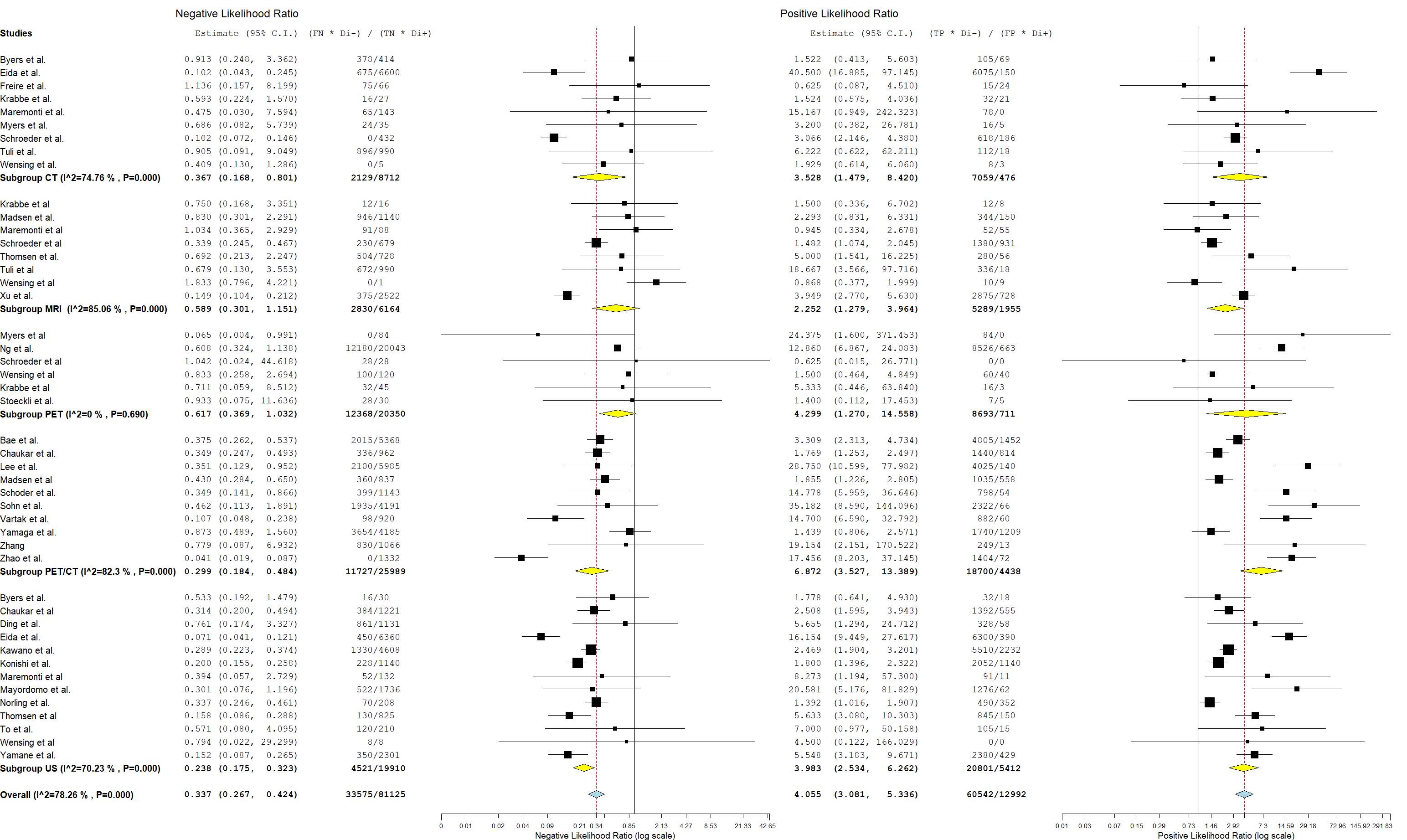

Supplement: Supplementary file 1 [file jcm-13-07622-s001.zip › Supplementary Figure S5.png]

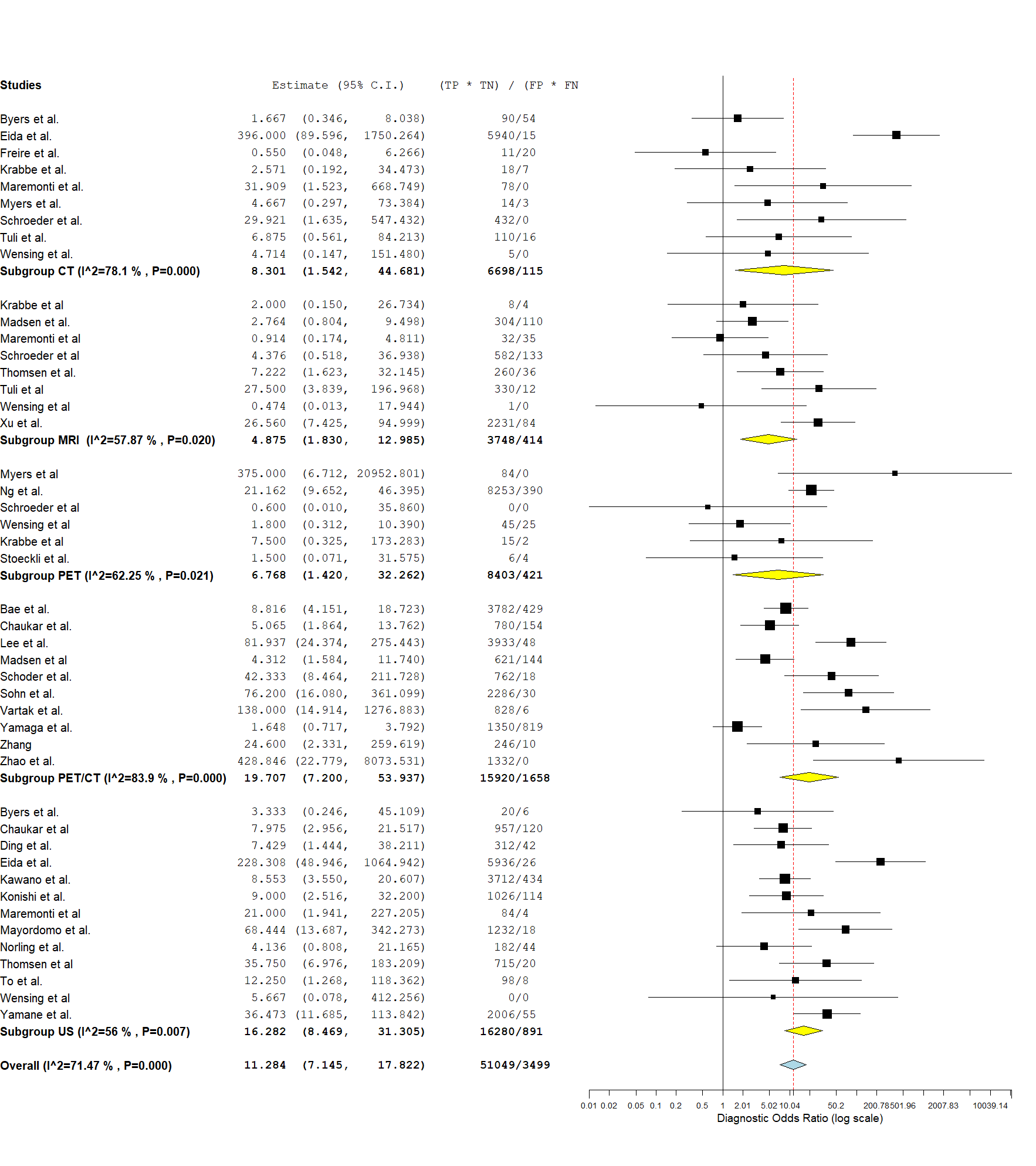

Supplement: Supplementary file 1 [file jcm-13-07622-s001.zip › Supplementary Figure S6.png]
